# Senolytic therapy is neuroprotective and improves functional outcome long-term after traumatic brain injury in mice

**DOI:** 10.3389/fnins.2023.1227705

**Published:** 2023-07-27

**Authors:** Jing Wang, Yujiao Lu, Christopher Carr, Krishnan M. Dhandapani, Darrell W. Brann

**Affiliations:** Department of Neurosurgery, Medical College of Georgia, Augusta University, Augusta, GA, United States

**Keywords:** traumatic brain injury, senescent cell, senolytic drug, astrocyte, microglia, inflammation

## Abstract

**Introduction:**

Chronic neuroinflammation can exist for months to years following traumatic brain injury (TBI), although the underlying mechanisms remain poorly understood.

**Methods:**

In the current study, we used a controlled cortical impact mouse model of TBI to examine whether proinflammatory senescent cells are present in the brain long-term (months) after TBI and whether ablation of these cells via administration of senolytic drugs can improve long-term functional outcome after TBI. The results revealed that astrocytes and microglia in the cerebral cortex, hippocampus, corpus callosum and lateral posterior thalamus colocalized the senescent cell markers, p16^Ink4a^ or p21^Cip1/Waf1^ at 5 weeks post injury (5wpi) and 4 months post injury (4mpi) in a controlled cortical impact (CCI) model. Intermittent administration of the senolytic drugs, dasatinib and quercetin (*D* + *Q*) beginning 1-month after TBI for 13 weeks significantly ablated p16^Ink4a^-positive- and p21^Cip1/Waf1^-positive-cells in the brain of TBI animals, and significantly reduced expression of the major senescence-associated secretory phenotype (SASP) pro-inflammatory factors, interleukin-1β and interleukin-6. Senolytic treatment also significantly attenuated neurodegeneration and enhanced neuron number at 18 weeks after TBI in the ipsilateral cortex, hippocampus, and lateral posterior thalamus. Behavioral testing at 18 weeks after TBI further revealed that senolytic therapy significantly rescued defects in spatial reference memory and recognition memory, as well as depression-like behavior in TBI mice.

**Discussion:**

Taken as a whole, these findings indicate there is robust and widespread induction of senescent cells in the brain long-term after TBI, and that senolytic drug treatment begun 1-month after TBI can efficiently ablate the senescent cells, reduce expression of proinflammatory SASP factors, reduce neurodegeneration, and rescue defects in reference memory, recognition memory, and depressive behavior.

## Introduction

Traumatic brain injury (TBI) is a major cause of long-term disability in the United States (Maas et al., 2017). While TBI is induced by an acute trauma, there is increasing evidence that it is a chronic and progressive disorder (Loane et al., 2014; Simon et al., 2017; Xiong et al., 2018). It has been proposed that over the long-term, the initial injury spreads via neuroinflammatory signals to nearby healthy regions - thereby amplifying the original focal tissue injury (Johnson et al., 2013; Loane et al., 2014). Emerging studies indicate that chronic and progressive neuroinflammation involving release of proinflammatory cytokines may contribute significantly to neurodegeneration after TBI, which may be treatable long after the initial brain injury (Li et al., 2009; Faden et al., 2016; Xiong et al., 2018).

The mechanisms underlying chronic neuroinflammation have been an area of significant interest. Recently, evidence has accumulated that proinflammatory “senescent” cells accumulate in the brain following injury, aging and in neurodegenerative disorders (Baker et al., 2011; Chinta et al., 2015; Baker and Petersen, 2018; Tominaga et al., 2019; Zhang et al., 2019). Senescent cells are cells that are damaged, have ceased to divide, are resistant to dying, and release proinflammatory cytokines such as interleukin-1β (IL-1β) interleukin-6 (IL-6) and tumor necrosis factor-alpha (TNF-α), proteases and growth factors that induce chronic inflammation and damage neighboring cells (Baker et al., 2011; Tchkonia et al., 2013). This secretory phenotype of senescent cells has been termed the “Senescence Associated Secretory Phenotype” (SASP) (Tchkonia et al., 2013; Baker and Petersen, 2018). Senescent cells can be identified by several hallmarks, including cell cycle arrest and over-expression of cell cycle inhibitors [p21^Cip1/Waf1^ (p21), p16^Ink4a^ (p16), p19^Ink4d^ (p19)], flattened morphology, DNA damage foci, presence of γH2AX (a histone variant that is phosphorylated in response to DNA damage), decreased lamin B1 expression, and secretion of SASP factors (Debacq-Chainiaux et al., 2009; Childs et al., 2017; Baker and Petersen, 2018).

Senescent cells have been observed in many tissues of the body, including the brain, and are significantly increased in aging. Intriguingly, genetic, or pharmaceutical ablation of senescent cells by administration of senolytic drugs such as the prototypical dasatinib and quercetin (*D* + *Q*), has been shown to extend the life span and health span in animals (Baker et al., 2011, 2016). In the brain, astrocytes and microglia can become senescent and contribute to both aging and neurodegenerative disorders such as TBI, Alzheimer’s disease (AD), and Parkinson’s disease (PD) (Salminen et al., 2011; Baker and Petersen, 2018; Kritsilis et al., 2018; Cohen and Torres, 2019; Schwab et al., 2019; Tuzer and Torres, 2022; Ng et al., 2023). With respect to TBI, controlled cortical impact (CCI) in young and old mice was found to induce elevated DNA damage and expression of senescent markers in microglia in the injured brain at 72 h post injury (Ritzel et al., 2019). Furthermore, examination of postmortem brains from professional athletes who had multiple mild TBIs also revealed increased DNA damage and expression of senescence markers and SASP factors (Schwab et al., 2019). Repeated mild TBI in mice similarly showed increased DNA damage markers at 24 h and increased expression of p21, P53, and the SASP, IL-1β in the injured cortex at 7 days (Schwab et al., 2021). Interestingly, administration of the senolytic drug ABT263 at 1 week after repeated TBI significantly improved performance on the Morris water maze when examined 1-week later (Schwab et al., 2022).

While these studies demonstrate that senescent cells are present acutely after TBI, the studies primarily focused on acute time points. Thus, the long-term temporal pattern of senescent cell accumulation in the brain after TBI has not been carefully studied. Furthermore, since many patients are long past the time of their TBI, it would be important to determine whether senolytic therapy can be started long-term after TBI and still yield functional benefit. Therefore, the purpose of the current study was to determine whether senescent cells are present in the brain weeks to months after TBI in mice, and whether ablation of these cells via administration of senolytic drugs beginning 1-month after TBI can improve long-term functional outcome after TBI.

## Materials and methods

### Animals

Adult male 6-month-old C57BL/6 J mice were obtained from Jackson Lab (Bar Harbor, ME) for use in this study. Mice were placed in the same room for at least 72 h before experiments and were housed under humidity and temperature-controlled conditions with free access to food and water. All animal experiments were approved by the Augusta University Institutional Animal Care and Use Committee.

### Controlled cortical impact

Controlled cortical impact (CCI) or sham surgery was performed on mice as described previously (Laird et al., 2010; Zhang et al., 2012; Ma et al., 2017; Wang et al., 2017). Isoflurane (2–4%) was used to anesthetize mice, which were then placed in a stereotaxic frame (Leica Impact One Stereotaxic Impactor, Buffalo Grove, IL, USA) for CCI or sham surgery. A 3.5 mm craniotomy was made in the right parietal bone midway between the lambda and the bregma with the medial edge 1 mm lateral from the midline. The dura was left intact. TBI mice were impacted using a 3 mm diameter convex tip (4.5 m/s, 20 ms dwell time, 1 mm depression) to produce a moderate TBI. Bone wax covered the cranial window following impact, and surgical staples closed the scalp incision. Mice were allowed to recover before being placed back into their housing environment. Body temperature was monitored and maintained at 37°C using a small thermometer (Kopf Instruments, Tujunga, CA, USA) throughout the operation. Sham operated mice received identical treatment except there was no impact. Power analysis (*α* = 0.05, *β* = 0.02) and experience indicate that a sample size of *n* = 7–12 is sufficient to overcome viability and ensure statistical difference. For each experiment, we initially started with *n* = 10 animals per group. Animals which were sick, failed to survive after surgery, or showed weight loss over 15%, were excluded from the study.

### Senolytic treatment

The senolytic drugs, dasatinib (Sigma, CDS023389-25MG, 5 mg/kg per day) plus quercetin (Sigma, Q4951-10G, 50 mg/kg per day), or Vehicle (VH, 10% DMSO) were administered at 1-month post-TBI by oral gavage for 3 consecutive days a week for 13 weeks (Saccon et al., 2021). Six-month old adult male C57BL/6 J mice were used and randomly divided into four groups: Sham + VH treatment; Sham + D&Q treatment; TBI + VH treatment; TBI + D&Q treatment. Vehicle treatment groups received equal volume of placebo as senolytic treatment groups.

### Barnes maze test

The Barnes Maze test was conducted to evaluate the hippocampal-dependent spatial reference memory, as previously described (Lu et al., 2020). Briefly, the test was divided into two stages: training trial (day 1 – day 3) and probe trial (day 4). One hole on the platform was set as the target hole with a hidden chamber placed beneath it. At each day of training stage, mice were initially put in the center of the platform to start exploring. The test was stopped when mice entered the target hole, and each mouse was allowed a maximal 3 min to explore. At the probe stage, the hidden chamber was removed, and the target hole was blocked with a black board. The escape latency, exploring time in the target quadrant and escape speeds during a 90-s period were recorded with a camera overhead, which was controlled by ANY-maze video tracking software (Stoelting Co., Wood Dale, IL). All tracking plots and parameters were analyzed after the experiment.

### Novel object recognition test

The novel object recognition test was performed to assess hippocampal-dependent recognition memory (Lu et al., 2020). This task was divided into a sampling stage (day 1) and a choice stage (day 2). Briefly, 1 day before the test, mice were put in the empty arena (56 cm * 56 cm * 45 cm) for 5-min of free exploration for habituation. At the sampling stage, two identical objects were placed in the box with equal distance, and mice were then put inside for a 5-min exploration period. Twenty-four hours later at the choice stage, one of the two objects was replaced with a novel object of identical size, but different shape and appearance. The exploring activity was recorded with ANY-maze video tracking software (Stoelting Company). Exploration time on each object and the discrimination index (the percentage of time spent exploring the novel object) were analyzed afterwards with ANY-maze software. Object exploration is defined as the mouse’s nose being within 2 cm’s range from the object.

### Forced swim test

The forced swim test was conducted to evaluate depressive-like states and behavioral despair, as described in our previous study (Lu et al., 2017, 2019). Briefly, mice were placed in a 2 L beaker filled with water of 25 ± 1°C. The water level was set as 4.5 cm from the top to make sure mice could not touch the bottom with their tails, or escape from the top. During a 6-min test period, the immobility time of each mouse was recorded. Immobility is defined as lack of movement except that required to keep the mouse afloat and breathe. After each test, the mice were gently warmed under a heating lamp, and returned to their home cages.

### Preparation of brain sections and histological analysis

Mouse brain sections were prepared as previously described (Lu et al., 2019; Wang et al., 2020). Briefly, mice were transcardially perfused at times indicated in the figure legends with ice-cold 0.9% saline followed by 4% paraformaldehyde under deep anesthesia. Mice were then decapitated, and brain tissues were collected and put in 4% paraformaldehyde for post-fixing at 4°C for 48 h. Brain tissues were then transferred to a 30% sucrose solution for dehydration until they completely sunk. Afterwards, the brain tissues were embedded in tissue embedding compound (Fisher HealthCare, 4585), and cut into frozen-sections of 25-μm thickness in series on a Leica Rm2155 microtome. The following antibodies were used for histological analysis: anti-GFAP (Abcam, Cat# 53554, 1:800), anti-IBA1 (Abcam, Cat#ab5076, 1:400), Anti-p16 (Invitrogen, Cat# MA5-17142, 1:400), and Anti-p21 (Invitrogen, Cat# AHZ0422, 1:400). Fluorescent images were captured on an LSM510 Meta confocal laser microscope (Carl Zeiss), using 40× or 63× oil immersion Neofluor objectives with the image size set at 1024 × 1024 pixels. IntDen values were measured with ImageJ software. For unbiased comparison across different groups, IntDen measurements were normalized to the cell-free background. Cell counts were carried out manually. All analysis was conducted with unmodified images. At least 4–5 representative sections per animal were used for immunostaining, and the typical image was selected for presentation.

### Interleukin-6 (IL-6) enzyme-linked immunosorbent assay (ELISA)

The brain IL-6 levels were measured in 50 μL hippocampal tissue lysates using Quantikine ELISA Kits (R&D system, M600B) following the manufacturer’s instruction. Briefly, mouse IL-6 control and standard were freshly prepared before assay. 50 μL of Assay Diluent was added to the center of each well followed by 50 μL of standard, control, or sample. Mixed by gently tapping and incubate at room temperature for 2 h. After washing 4 times with a wash buffer, 100 μL of Mouse IL-6 conjugate was added into each well and incubated for 2 h. After washing 4 times, 100 μL of substrate solution was added to each well and incubated while protecting from light for 30 min at room temperature. After adding 100 μL of stop solution, the optimal density of each well was determined using a microplate reader at 450 nm, correcting by subtracting the reading value at 540 nm. The IL-6 levels were calculated by their concentrations in the lysate normalized to the protein concentration of each sample.

### Neuronal survival quantification and Fluoro Jade B staining

For immunohistochemistry (IHC) analysis, a total of 60 coronal sections were collected between −0.82 and − 2.54 mm from bregma. six sections from each animal, at even 8 section intervals. As previously described (Hoover et al., 2004; Thompson et al., 2006; Ma et al., 2017), lesion area was outlined using ImageJ freeform selection tool after cresyl violet staining. Sections were incubated with anti-NeuN (rabbit, 1:1000, Millipore, #ABN78) at 4°C overnight, then with freshly prepared Fluoro-Jade C solution (1:1000, Merck Millipore Corporation, catalog number: AG310) at room temperature for 15 min. NeuN^+^ and Fluoro Jade C^+^ cells were counted in a consistent and set area in the perilesional cortex using 40× confocal images. Set linear threshold and circularity index were kept as constant parameters across all analyzed images in ImageJ for automated cell counting. At least 3 sections from 4 mice per experimental group were examined for quantification of neuronal survival.

### Measurement of white matter damage

White matter damage was evaluated by measuring the immunoreactivity of myelin basic protein (MBP) with immunohistochemistry and Western blot. For histological analysis, brain sections were stained with anti-MBP (1:400, Cell Signaling, #78896), and the mean immunofluorescent intensity of MBP was quantified at corpus callosum (CC) as well as cortex. Brain lysate of cortical tissue from the perilesional area was used to measure the expression of MBP by Western blot. As MBP is expressed in multiple isoforms, total band intensity between 21 KD to 14 KD was quantified.

### Western blot analysis

Cortical tissue from the perilesional area (or an anatomically matched cortical area on sham mice) were collected at specified time points after TBI as previously described by our laboratory (Zhang et al., 2012; Ma et al., 2017; Wang et al., 2017). The isolated tissue was immediately frozen in liquid nitrogen or homogenized in ice-cold RIPA buffer (50 mM Tris–HCl pH 7.4, 150 mM NaCl, 1% Triton X-100, 1% sodium deoxycholate, 0.1% SDS, 1 mM EDTA; with Roche complete protease inhibitor and PhosphoSTOP) with a tissue tearor. The homogenate was centrifuged at 12,000 RPM for 15 min at 4°C, and the supernatant was aliquoted for further analysis. A BCA Protein Assay (Thermo Fisher Scientific, Carlsbad, CA) was used to determine protein concentrations. 20 μg samples of protein were separated on a 4–20% SDS-PAGE gel and transferred onto 0.2 μM nitrocellulose membranes. Blots were blocked with 5% nonfat milk for 1 h at room temperature with gentle shaking. After blocking, the blots were incubated overnight in 4°C with primary antibodies: Iba1 (goat, 1:1000, Abcam, #ab5076); p16 (Rabbit monoclonal to CDKN2A/p16INK4a, 1:1000, Abcam, #ab211542); p21 (Rabbit monoclonal, 1:1000, Abcam, #ab188224); IL-1β (Rabbit polyclonal, 1:1000, Abcam, #ab9722). GAPDH (mouse, 1:2000, Santa Cruz Biotechnology, #sc-32233) was used as a loading control. The membrane was then washed with 1× TBST 3 times and then incubated with secondary antibodies. Bound proteins were visualized using enhanced chemiluminescence (ECL), and band densities were quantified using Image Studio (LI-COR, Inc.).

### qRT-PCR

Total RNA was isolated from the ipsilateral cortex of sham and TBI mouse brains using the SV Total RNA isolation Kit (Promega) according to the manufacturer’s instruction. 400 ng of RNA was used for reverse-transcription polymerase chain reaction (RT-PCR) using the SuperScript III Platinum SYBR Green One-Step qRT-PCR Kit (Invitrogen, Grand Island, NY, USA). The reaction was performed with CFX Opus 96 Real-Time PCR System (BioRad). The cycle of threshold values was first normalized to multiple housekeeping genes encoding for UBC in the same sample. The relative transcript levels for each gene were then reported as “fold changes versus sham controls” by calculating the 2^−ΔΔCT^ value. The sequence of primers used are as follows (5′ to 3′, forward/reverse): CDKN2A (p16 gene) (TTGGCCCAAGAGCGGGGACA/GC GGGCTGAGGCCGGATTTA), CDKN1A (p21 gene) (TGAGCCGCC ACTGTGATG/GTCTCGGTGACAAAGTCGAAGTT), CDKN2D (p19 gene) (GGAGCTGGTGCATCCTGACGC/TGGCACCTTGCTTC AGGAGCTC), SERPINE1 (Pal-1 gene) (CCTCTTCCACAAGTCTGA TGGC/GCAGTTCCACAACGTCATACTCG), IL6 (TAC CACTTC ACAAGTCGGAGGC/CTGCAAGTGCATCATCGTTGTTC), IL1β (TGGACCTTCCAGGATGAGGACA/GTTCATCTCGGAGCCTGT AGTG), UBC (GTGTCTAAGTTT CCCCTTTTAAGG/TTGGGAATGCAACAACTTTATTG).

### Statistical analysis

GraphPad Prism 6 software was used to analyze all the data. Data are presented as mean ± SEM. Two-tailed unpaired Student t-test was performed when only comparing two groups (e.g., sham and TBI-4mpi groups). A one-way ANOVA test was conducted to analyze differences within sham and the different time points following TBI. Two-way ANOVAs were used to compare the difference between vehicle (VH) and *D* + *Q* treated animals in sham or after TBI. When the ANOVA test was found to be significant, the Tukey’s honestly significant difference posthoc test was conducted to make pairwise comparisons between experimental groups. A value of *p* < 0.05 was considered statistically significant. All studies were randomized, and all analyses were blinded.

## Results

### Evidence of progressive neurodegeneration in the TBI brain

**Figure 1:**
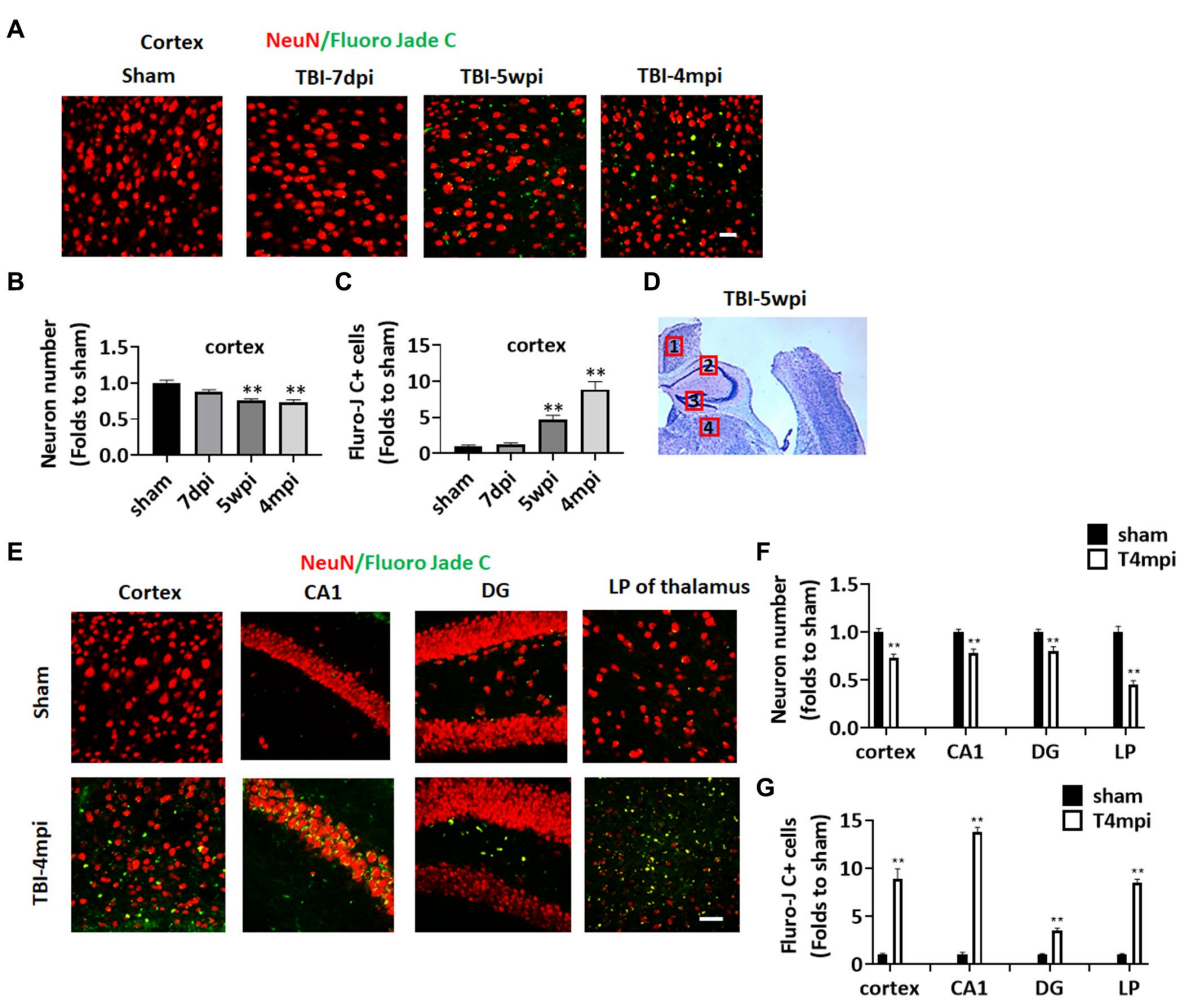
Progressive neuronal loss and damage at 5 weeks and 4 months after TBI. **(A)** Representative images of immunostaining for NeuN (red) and a neurodegeneration marker Fluoro Jade C (green) to detect neuronal damage in the ipsilateral injured cortex at 7-dpi (days post injury), 5-wpi (weeks post injury) or 4-mpi (months post injury). **(B,C)** Quantification of NeuN+ cells **(B)** and Fluoro-Jade C+ cell numbers **(C)** in the cortex. **p < 0.01 compared to sham. **(D)** Representative image of ipsilateral injured brain at 5-wpi by cresyl violet staining. Numbers show the neuronal damaged areas, where the representative images were taken: 1, cortex; 2, CA1; 3, DG; 4, LP of thalamus. **(E)** Representative images showing wide-spread Fluoro-Jade C+ neurons (Yellow dots) in various brain regions at 4mpi. **(F,G)** Quantification of NeuN+ cells **(F)** and Fluro-Jade C+ cell numbers **(G)** in different regions of the brain. Scale bars: 20 μm. Sham *n* = 7, TBI *n* = 8.

Progressive neurodegeneration is widely regarded as a hallmark of chronic neuroinflammation. We thus examined neurodegeneration in adult male mice at 7 days post injury (7dpi), 5 weeks post injury (5wpi) and 4 months post injury (4mpi). Sham animals were used as controls. Neuron loss and neurodegeneration were assessed in various brain regions by performing IHC for the neuronal marker, NeuN, and a neurodegenerative marker, Fluoro-Jade C (FJC), respectively (Figure 1). Quantification of NeuN+ cell number at the ipsilateral injured cortex (a cortical region adjacent to the lesion, e.g., area 1 in Figure 1D), demonstrated a progressive decrease in the neuron number from 5wpi to 4mpi (Figures 1A,C). The number of FJC+ cells were significantly increased in the same area over that same time period, indicating progressive neurodegeneration after TBI (Figures 1A,D). Interestingly, we observed wide-spread FJC+ neurons (Yellow dots in Figures 1E,F) in additional brain regions to the perilesional cortex [including the hippocampal CA1 region, dentate gyrus (DG), and lateral posterior thalamus (LP of Thalamus) at 4mpi]. These FJC+ neurons were not detected in the age-matched shams and showed only limited distribution in the perilesional regions at 7dpi and 5wpi (Figure 1A). These results demonstrate that there is progressive and widespread neurodegeneration after TBI.

### Evidence of a long-term cognitive defect after TBI

**Figure 2:**
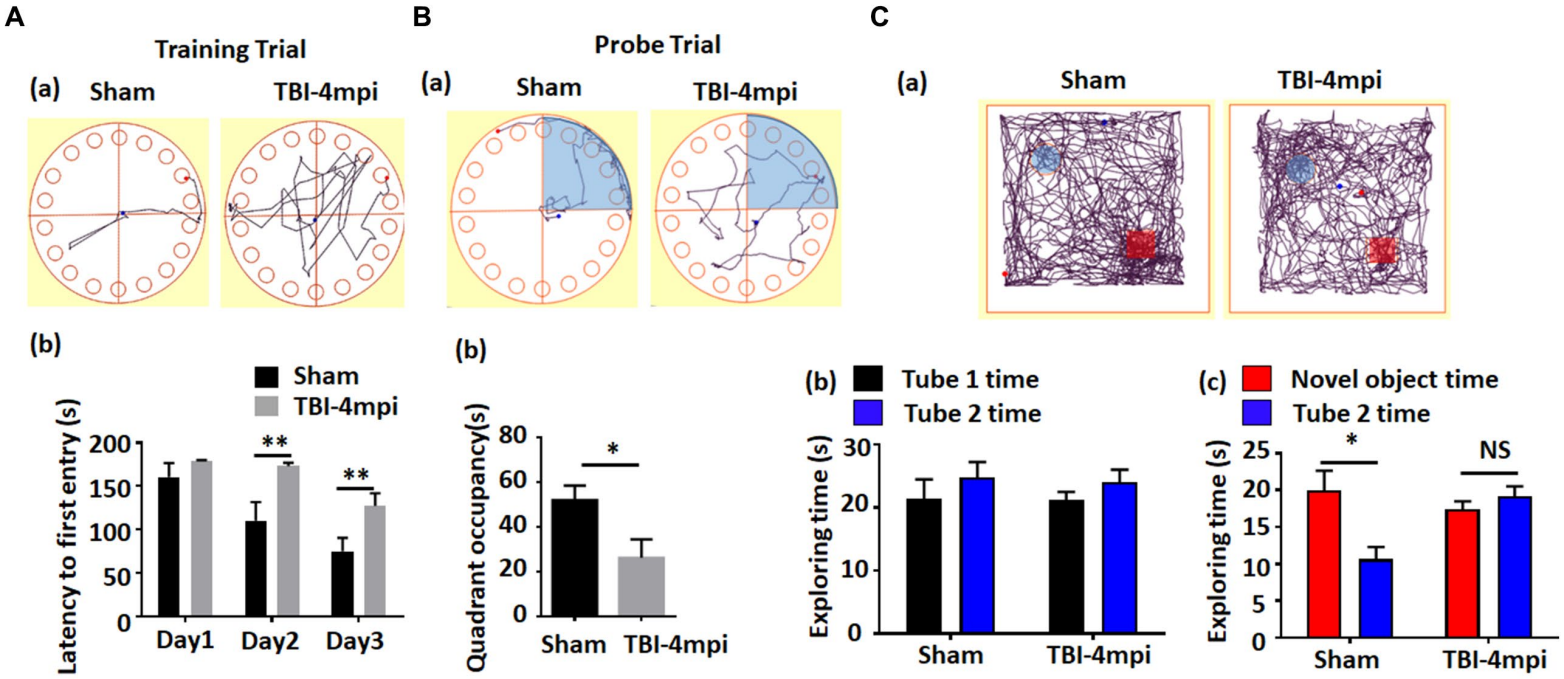
Ten-month old male mice exhibit a significant defect in learning and memory at 4 months after TBI. **(A)** Barnes Maze training trial shows a significant increase of escape latency in TBI-4mpi group at day 2 and day 3 in training **(b)**. **(a)** shows representative tracking plot of sham and TBI-4mpi mice at day 3 of Barnes Maze training trial. **(B)** Barnes Maze probe trial shows significant decrease of quadrant occupancy in TBI-4mpi group **(b)**. **(a)** shows representative tracking plot of sham and TBI-4mpi mice on Barnes Maze probe trail. **(C)** Novel Object Recognition test demonstrates that mice from the sham but not the TBI-4mpi group showed a significant decrease in exploring time on the familiar object on choice stage **(c)**. On sampling stage **(b)**, no significant difference in exploring time on two tubes was detected in both sham and TBI-4mpi groups. **(a)** shows representative tracking plot of sham and TBI-4mpi mice on test day. Sham *n* = 7, TBI *n* = 8. *p < 0.05. **p < 0.01. NS, not significant.

We next examined cognitive function in 10-month-old male mice at 4 months after TBI. The Barnes maze test was used to examine reference memory, while the Novel Object Recognition test (NOR) was used to examine recognition memory. As shown in Figure 2, TBI-4mpi mice showed a significant increase of the latency to first entry on days 2 and 3 in the training trial of the Barnes maze test as compared to Sham mice (Figures 2Aa,b), and a significant decrease of quadrant occupancy in the probe trial of the Barnes maze test as compared to Sham mice (Figures 2Ba,b), which indicates that TBI-4mpi mice have a significant defect in reference memory. We next examined hippocampal-dependent recognition memory using the NOR test. Representative tracking plots for the mice in the NOR test on test day (Choice stage) are shown in Figure 2Ca. Figure 2Cb shows that sham and TBI-4mpi mice have similar exploring time on two identical objects in the sampling stage, which indicates that the mice were not biased to the same objects located in different positions. However, in the choice stage, we observed a significantly reduced preference to the novel object in TBI-4mpi mice as compared with Sham mice (Figure 2Cb), indicating that there is also significant impairment in recognition memory in mice 4 months after TBI.

### Evidence of a chronic neuroinflammatory response in the TBI brain

**Figure 3:**
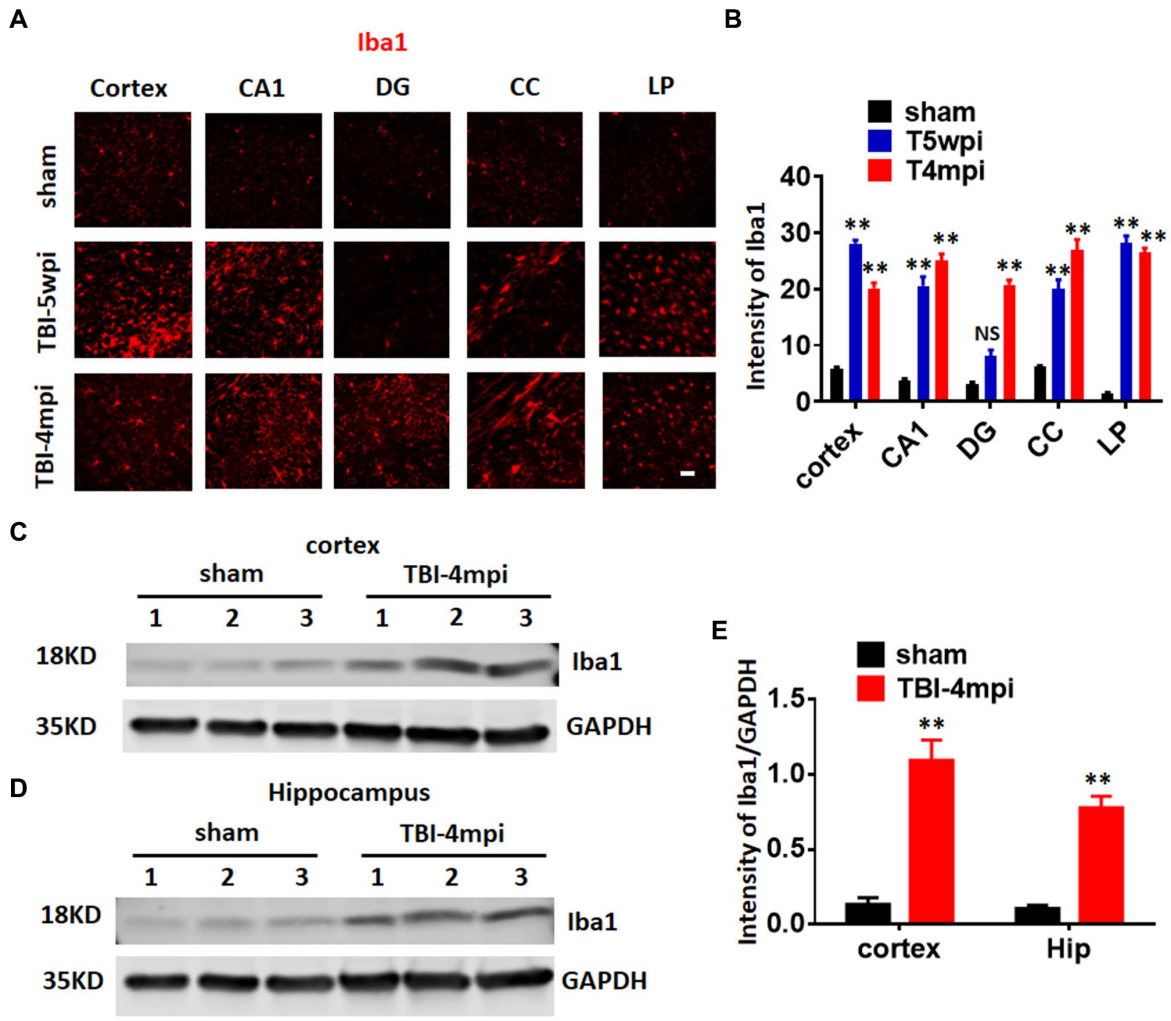
A chronic and widespread inflammatory response is present at 4-month post injury (mpi) after TBI. **(A)** Representative images showing wide-spread microglia reactivation (red, stained by anti-Iba1 in various brain regions at 5wpi and 4mpi). DG, dentate gyrus; CC, corpus callosum; LP, lateral posterior nucleus. Scale bar: 20 μm. **(B,D)** Quantification of immunofluorescence (IF) intensity of Iba1. **(C,D)** Western blot detected enhanced Iba1 expression in the cortex **(C)** and hippocampal **(D)** tissue. **(E)** Quantification of Western blot on Iba1. **p < 0.01 compared to sham. Sham *n* = 7, TBI *n* = 8. NS, not significant.

Microglia are well known to play an important role in mediating neuroinflammatory processes in the brain after injury. Therefore, we next examined microglial activation in the TBI brain at 5wpi and 4mpi. The expression and intensity of the microglial marker, Iba1, was used as an indicator of microglial activation. As shown in Figures 3A,B, immunostaining of brain sections for Iba1 revealed robust and widespread microglia activation in the perilesional cortex, hippocampal CA1 region (CA1), DG, corpus callosum (CC), and LP of Thalamus at 5wpi and/or 4mpi. Western blot analysis further confirmed robust Iba1 protein expression in the cortex and hippocampus at 4mpi (Figures 3C–E). These findings demonstrate a widespread neuroinflammatory response long-term after TBI.

### Evidence of long-term white matter damage after TBI

**Figure 4:**
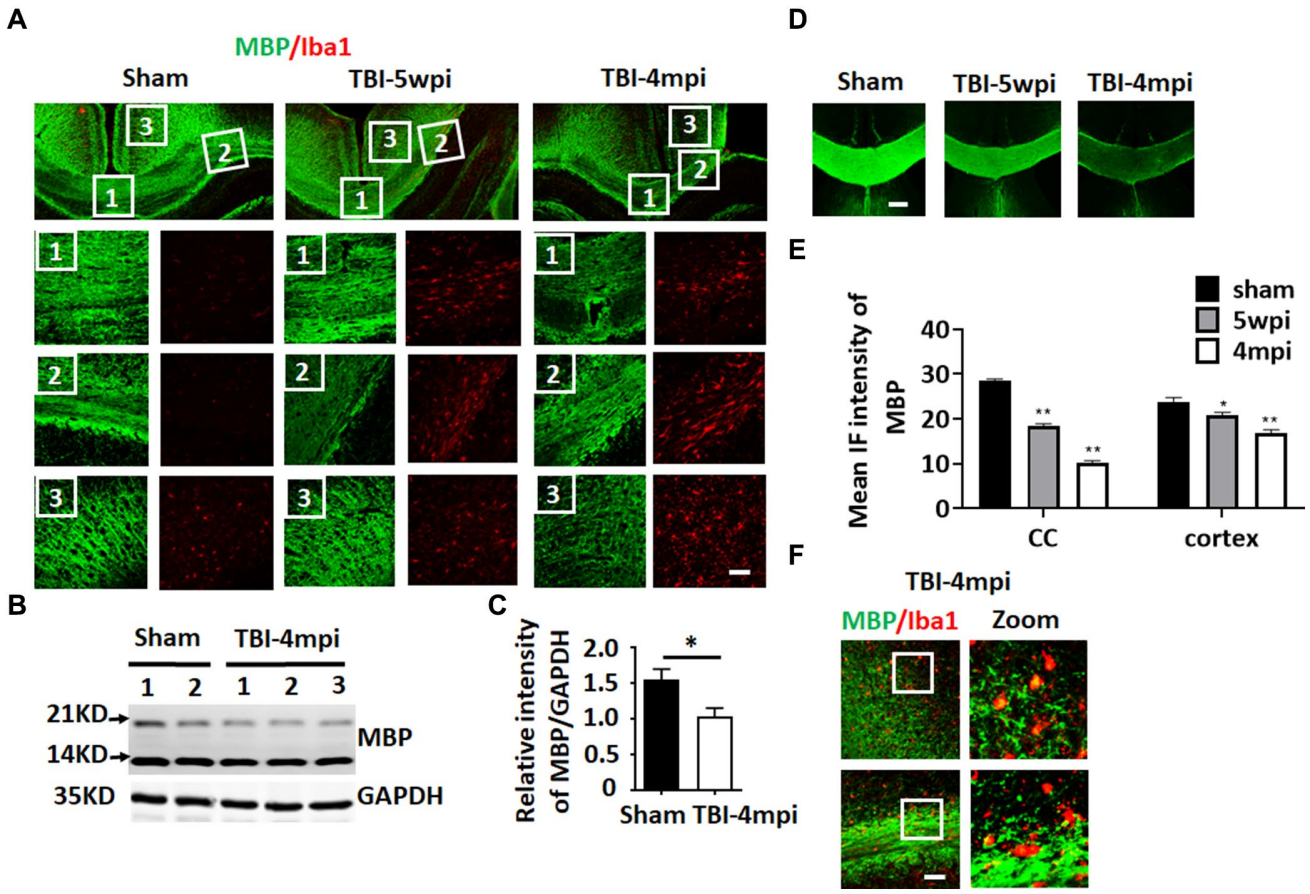
Progressive and wide-spread white matter damage at 4 months after TBI. **(A)** Representative image of immunostaining for MBP (green) and Iba1 (red) to detect myelin distribution and microglia activation in sham, TBI-5wpi and TBI-4mpi mouse brain. Scale bar: 40 μm. **(B,C)** Western blot detected reduced MBP expression in ipsilateral cortex at 4mpi. **(D,E)** Decreased intensity of MBP at 5wpi and 4mpi. *p < 0.05, **p < 0.01 compared to sham. Scale bar: 80 μm. **(F)** Confocal microscopy showing co-localization of MBP inside Iba1-positive cells in TBI-4mpi mouse brain, indicating that microglia have taken up damaged myelin at the 4mpi time point. Scale bar: 20 μm. Sham *n* = 7, TBI *n* = 8.

Chronic neuroinflammation and microglial activation is known to be associated with white matter damage. Therefore, we next examined whether the TBI mouse brain shows any evidence of white matter damage long-term after TBI. To assess white matter damage, we performed IHC for myelin basic protein (MBP) and Iba1 (the microglial marker) on sham, TBI 5wpi and TBI 4mpi brain sections (Figure 4). Compared with sham mice, distribution of MBP in the TBI 5wpi and 4mpi mouse brains displayed an un-even and fragmented pattern in the CC (Figure 4A, zoomed 1 and 2) and ipsilateral injured cortex (Figure 4A, zoomed 3), indicative of white matter damage. Western blot analysis for MBP also revealed a significant decrease of MBP levels in the ipsilateral injured cortex at 4mpi (Figures 4B,C). Furthermore, MBP intensity in the CC and cortex regions demonstrated a significant reduction in the TBI 5wpi and 4mpi mouse brains (Figures 4D,E). Confocal microscopy further demonstrated co-localization of MBP within Iba1+ cell bodies in TBI-4mpi sections (Figure 4F, yellow dots in zoomed area), suggesting that activated microglia have taken up damaged myelin in the CC at 4 mpi. These findings demonstrate progressive white matter damage persists long-term after TBI, which is another hallmark of chronic neuroinflammation.

### Evidence of senescent cells in the brain long-term after TBI

**Figure 5:**
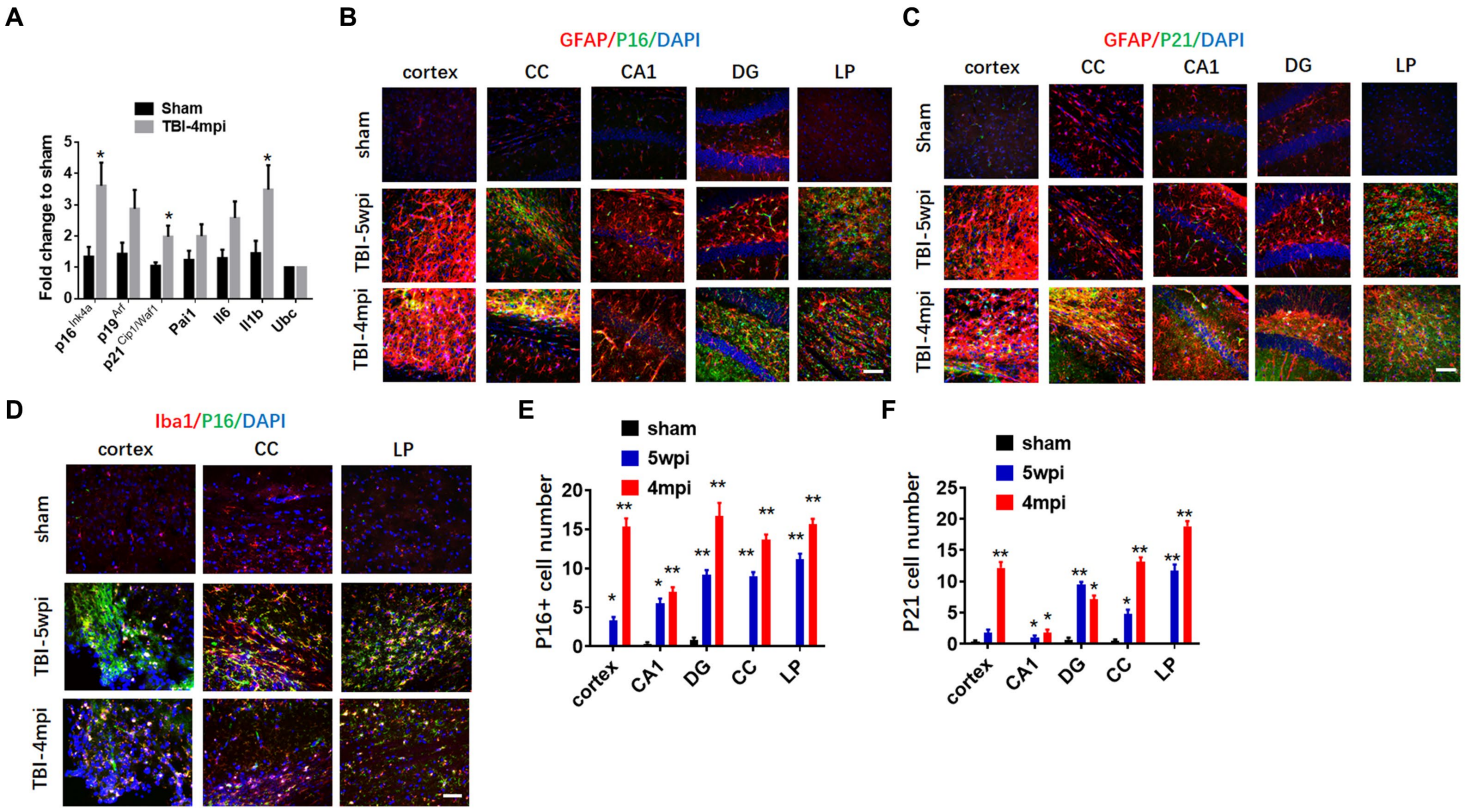
Astrocytes and microglia in TBI-5wpi and TBI-4mpi mouse brain express senescent cell markers. **(A)** qRT-PCR analysis of senescent indicators in cortex at 4 months after TBI. Data are Mean ± SEM, *n* = 8 mice. *p < 0.05 versus sham. **(B,C)** Representative image of immunostaining for the senescent cell markers p16 (**B**, green) and p21 (**C**, green) with GFAP (glial fibrillary acidic protein, red) in various brain regions at sham, 5wpi and 4mpi. Note the yellow color indicates localization of senescent cell marker in astrocytes. **(D)** Representative image of immunostaining for the senescent cell markers p16 (green) microglial marker Iba1 (red) in Cortex, CC and LP at 5wpi and 4mpi. Note the yellow color indicates localization of senescent cell marker in microglia. **(E,F)** Quantification of senescent cell markers p16 and p21 positive cell number in sham, 5wpi and 4mpi mouse brain. *p < 0.05, **p < 0.01 compared to sham. For histological study, Sham *n* = 7, TBI *n* = 8.

We next examined the expression of senescent cell markers in the cortex at 4mpi. qRT-PCR analysis of senescent cell markers in the mouse cortex at 4 months after TBI revealed significant up-regulation of several key markers of cellular senescence as compared to Sham mice, including p16, p21, and IL-1β. We next examined whether senescent cell markers are expressed in astrocytes and microglia in various brain regions long-term after TBI. We first examine astrocyte expression by performing double IHC for the astrocyte marker, GFAP, and either the senescent markers, p16 or p21 at 5wpi and 4mpi after TBI. As shown in Figures 5B,C, the IHC results demonstrate robust and widespread colocalization of both p16 and p21 in astrocytes (yellow color) at 5wpi, with an even greater colocalization noted at 4mpi in the cortex, CC, DG and LP of thalamus. Note that Sham animals show little to no colocalization of senescent markers in astrocytes in the various brain regions. Furthermore, semi-quantification of the IHC results shows highly significant increases of p16+ and p21+ cells in almost all of the brain regions, with significant elevation at 5wpi, followed by a progressive and even higher elevation of senescent cells at 4mpi (Figures 5E,F). Our studies also showed strong colocalization of the senescent marker p16^Ink4a^ in microglia in the cortex, CC, and LP of thalamus, indicating that many microglia are also senescent in the brain long-term after TBI (Figure 5D).

### Senolytic drug therapy ablates senescent cells and reduces pro-inflammatory SASP levels in the brain long-term after TBI

**Figure 6:**
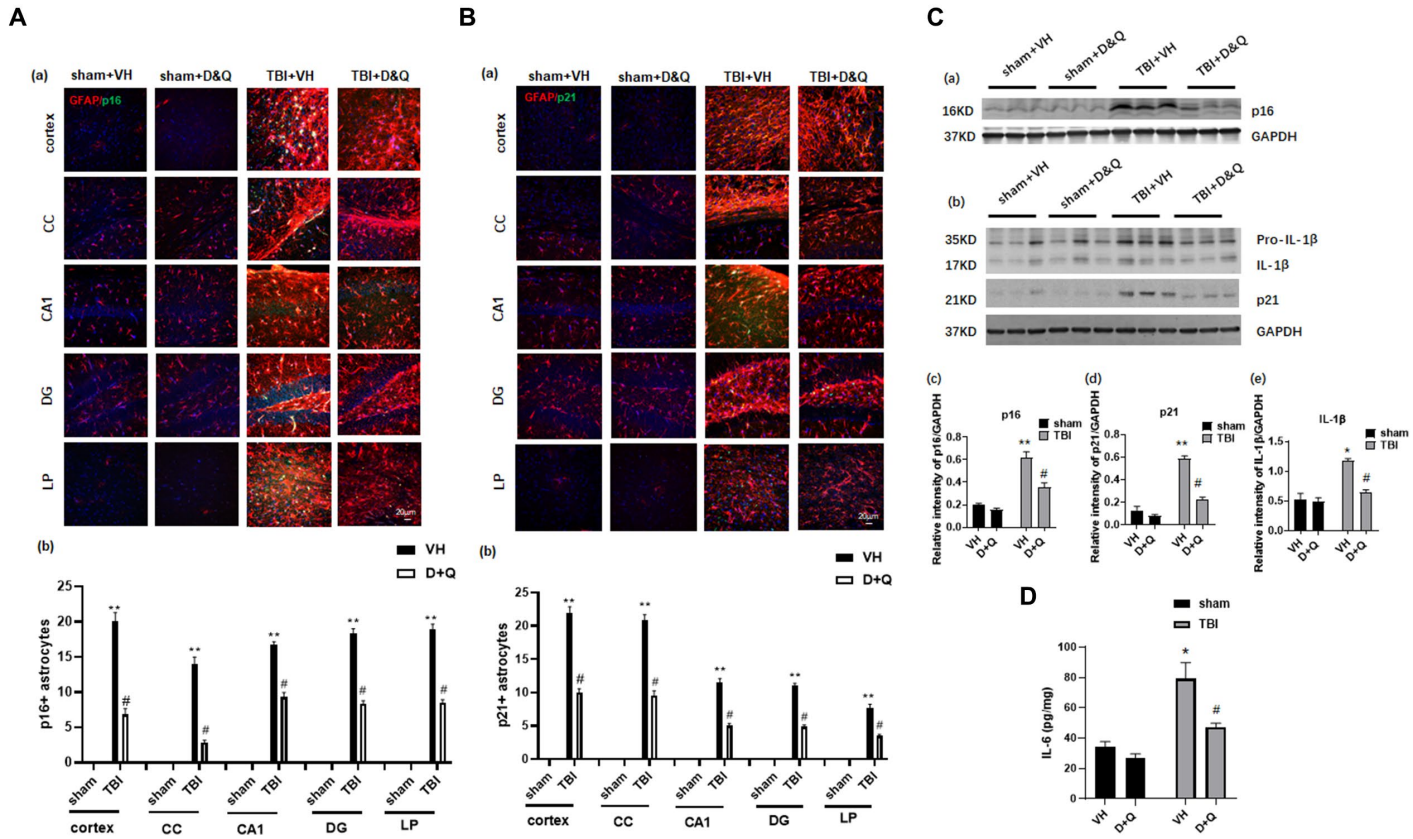
Senolytic therapy ablates senescent astrocytes and suppresses senescent cell marker expression after TBI. **(Aa)** Representative p16 and GFAP staining in cortex, CC, CA1, DG and LP of Thalamus in of the various groups at 18 weeks after TBI, and **(b)** quantification of p16^+^ astrocytes. **(Ba)** Representative p21 and GFAP staining in cortex, CC, CA1, DG and LP of Thalamus in of the various groups at 18 weeks after TBI, and **(b)** quantification of p21^+^ astrocytes. **(Ca,b)** Western blot of p16, p21, and IL-β in the cortex of the various groups at 18 weeks after TBI. **(c–e)** Quantification of the relative intensity of p16, p21, and IL-β to GAPDH. **(D)** ELISA measurement of IL-6 levels in brain lysates from the cortex of various groups at 18 weeks after TBI. ^*^p < 0.05, ^**^p < 0.01 vs. Sham+VH, ^#^*p* < 0.05 vs. TBI + VH. *n* = 8 for each group.

We next examined whether administration of the senolytic drugs, dasatinib and quercetin (*D* + *Q*) can ablate senescent cells and reduce levels of the pro-inflammatory SASPs IL-1β and IL-6 in the brain long-term after TBI. Beginning 1-month after TBI, Vehicle (VH) or *D* + *Q* (*D* = 5 mg/kg per day, *Q* = 50 mg/kg per day) was administered to male rats by oral gavage 3 consecutive days a week for 13 weeks. The effect of *D* + *Q* treatment on senescent cell burden was determined by double-IHC for the astrocyte marker GFAP and either p16 or p21, as well as Western blot analysis for expression of the senescent cell markers, p16 and p21. The effect of *D* + *Q* treatment on pro-inflammatory SASP burden in the ipsilateral cortex was measured by Western blot analysis of cortical tissue samples for IL-1β and by ELISA of cortical samples for IL-6. Representative IHC photomicrographs for GFAP with p16 and P21 are shown in Figures 6Aa,Ba, respectively, while semi-quantitative analysis of p16 and p21 intensity is shown in Figures 6Ab,Bb. As shown in Figures 6Aa,Ba, IHC for the astrocyte marker GFAP (red color) and the senescent cell markers p16 and p21 (green color) revealed significant colocalization of p16 and p21 in astrocytes in the TBI + VH brain in all regions examined. Note that Sham+VH and Sham + *D* + *Q* mice had low to no colocalization of GFAP and p16 or p21 in the various brain regions, and that *D* + *Q* in TBI mice dramatically reduced the number of p16^+^ and p21^+^ astrocytes in the various brain regions (Figures 6Aa,Ba). Semi-quantitative analysis of p16^+^ and p21^+^ astrocytes further confirmed low levels of p16^+^ and p21^+^ astrocytes in Sham+VH and Sham+*D* + *Q* mice, a robust elevation of p16^+^ and p21^+^ astrocytes in TBI + VH mice, and significant and dramatically reduced p16^+^ and p21^+^ astrocytes in all brain regions in *D* + *Q* + TBI mice as compared to the TBI + VH group (Figures 6Aa,b,Ba,b).

Western blot analysis of cortical samples further confirmed low expression of p16 and p21 expression in Sham+VH and Sham+*D*&*Q* mice, robust elevation in TBI + VH mice, and significant attenuation of p16 and p21 expression by *D* + *Q* treatment in TBI mice (Figures 6Ca–d). Western blot analysis also revealed that expression of the pro-inflammatory SASP factor IL-1β exhibited the same expression pattern as described for p16 and p21 expression in the cortex, with low expression in Sham+VH and Sham+*D*&*Q* mice, marked elevation observed in TBI + VH mice, and significant attenuation by *D* + *Q* treatment in TBI mice (Figures 6Ca,b,e). Likewise, ELISA of cortical samples for the proinflammatory SASP 1 L-6 revealed low levels of IL-6 in the cortex of Sham+VH and Sham+*D*&*Q* mice, marked elevation in TBI + VH mice, and significant attenuation by *D* + *Q* treatment in TBI mice (Figures 6Ca,b,e).

**Figure 7:**
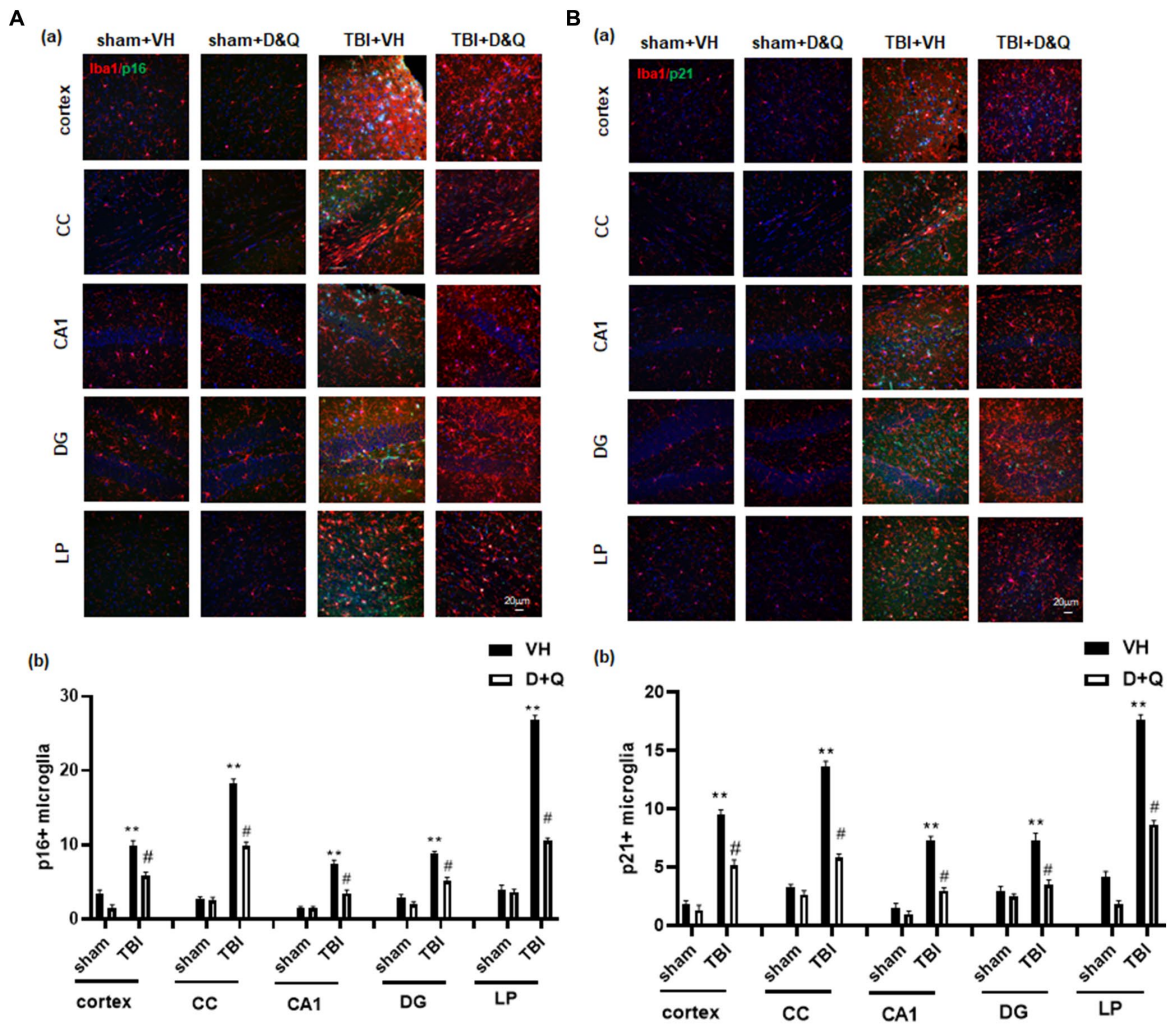
Senolytic therapy ablates senescent microglia after TBI. **(Aa)** Representative p16 and Iba1 staining in cortex, CC, CA1, DG and LP of Thalamus in of the various groups at 18 weeks after TBI, and **(b)** quantification of p16+ microglia. **(Ba)** Representative p21 and Iba1 staining in cortex, CC, CA1, DG and LP of Thalamus in of the various groups at 18 weeks after TBI, and **(B)** quantification of p21+ microglia. ^**^p < 0.01 vs. Sham+VH, ^#^*p* < 0.05 vs. TBI + VH. NS: no significant. Scale bar: 20 μm. *n* = 8 for each group.

We next examined *D* + *Q* effect upon ablation of senescent microglia cells in the brain long-term after TBI. As shown in Figures 7Aa,b, IHC for the microglial marker Iba1 (red color) and the senescent cell markers p16 and p21 (green color) revealed significant colocalization of p16 and p21 in microglia in the TBI + VH brain in all regions examined. Note that Sham+VH and Sham+*D* + *Q* mice had low to no colocalization of microglia and p16 or p21 in the various brain regions, and that semi-quantitative analysis revealed that *D* + *Q* in TBI mice dramatically reduced the number of p16+ and p21+ microglia in the various brain regions (Figures 7Aa,b).

### Senolytic drug therapy improves functional outcome long-term after TBI

**Figure 8:**
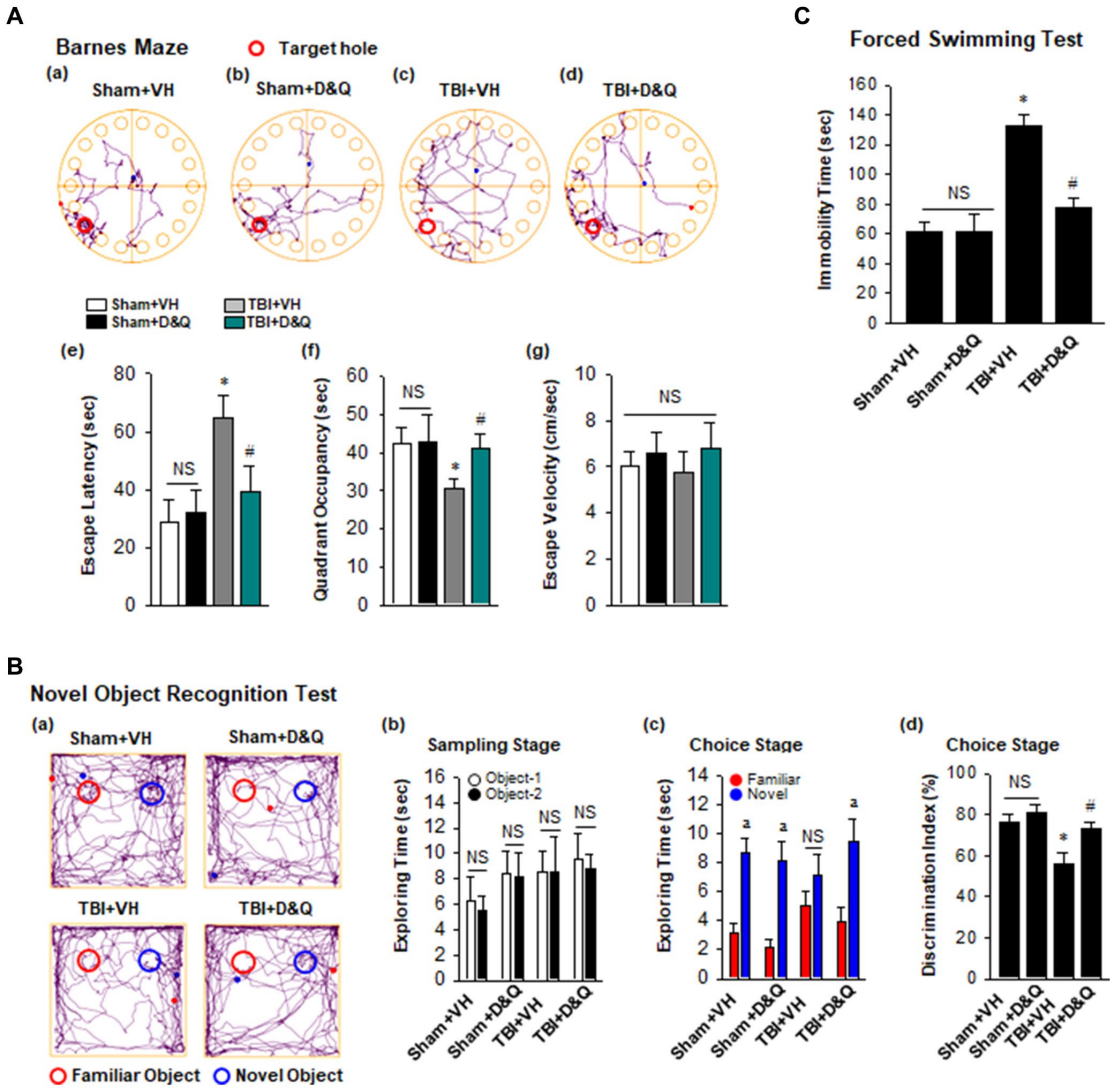
Senolytic therapy rescues cognitive function and depressive behavior after TBI. **(A)** Barnes Maze task was utilized to assess the spatial reference memory. Representative tracking plots of different groups on the probe trial were recorded **(a–d)**. Primary escape latency **(e)**, quadrant occupancy **(f)**, and escape velocity **(g)** during probe trial were analyzed. **(B)** Novel Object Recognition Test (NOR) was used to measure recognition memory. Representative tracking plots of indicated groups on NOR choice day are indicated **(a)**. Sampling stage exploring time **(b)**, Choice stage exploring time **(c)** and discrimination index **(d)** were statistically analyzed. **(C)** Forced swimming test was performed to examine depressive behavior. Values are means ± SEM of determinations from each group. ^*^p < 0.05 vs. Sham+VH, ^#^*p* < 0.05 vs. TBI + VH. ^a^*p* < 0.05 vs. Familiar object. NS, not significant. *n* = 8 for each group.

Figure 8A shows the result of the Barnes maze test, which tests spatial reference memory. Figures 8Aa–d shows representative tracking plots during the probe trial for all groups. Examination of TBI + VH animals at 18 weeks after TBI revealed that they had significant impairment of spatial reference memory, as evidenced by a significant increase in escape latency (Figure 8Ae) and decreased time in the quadrant where the escape hole was located (Figure 8Ae). In contrast, *D* + *Q* treatment begun 1-month after TBI significantly rescued the defect in spatial reference (Figures 8Aa–f). Note that *D* + *Q* had no effect in sham mice (Figures 8Aa–f). Also note that there was no difference in escape velocity between any of the groups (Figure 8Ag), indicating that the observed differences were not due to differences in motor function. We next used the NOR test to examine ability of *D* + *Q* to rescue the recognition defect in TBI mice. Representative tracking plots for the mice in the NOR test on the test day (choice stage) are shown in Figures 8Ba,b shows that all mice had similar exploring time on two identical objects in the sampling stage, which indicates that the mice were not biased to the same objects located in different positions. However, in the choice stage, TBI + VH mice had significantly reduced preference to the novel object as compared with Sham mice (Figures 8Bc,d), indicating significant impairment in recognition memory in mice 4 months after TBI, and this effect was significantly reversed by *D* + *Q* treatment of TBI mice. Furthermore, performance of the Forced Swim test revealed that TBI + VH mice had a significant increase in depressive-like behavior as compared to sham mice, and *D* + *Q* treatment was able to significantly reverse the depressive-like behavior in TBI mice (Figure 8C).

### Senolytic drug therapy reduces neurodegeneration and neuronal loss after TBI

**Figure 9:**
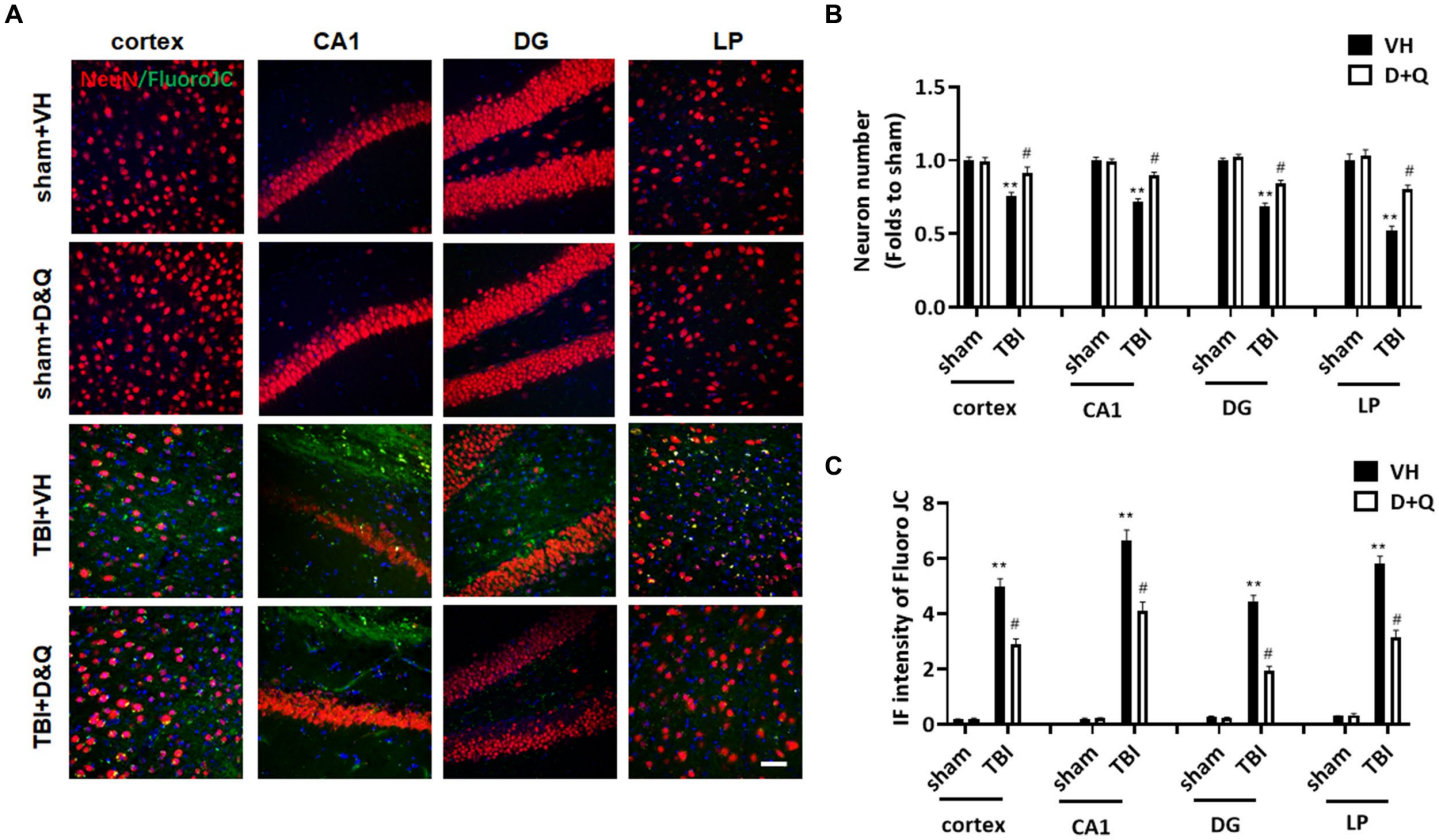
Senolytic therapy reduces neurodegeneration and neuronal loss after TBI. **(A)** Representative NeuN and FluroJC staining in cortex, CA1, DG and LP of Thalamus in of the various groups at 18 weeks after TBI, **(B)** Quantification of NeuN+ cell number. **(C)** Quantification of FluroJC staining. ^**^p < 0.01 vs. Sham+VH, ^#^*p* < 0.05 vs. TBI + VH. Scale bar: 20 μm. *n* = 8 for each group.

We next examined the effect of senolytic therapy on neuron loss and neurodegeneration in various brain regions by assessing NeuN and FJC staining, respectively. Figure 9A shows representative images of NeuN and FJC staining in cortex, CA1, DG and LP of Thalamus in the various groups at 18 weeks after TBI, while semi-quantitative analysis of NeuN cell number and FJC intensity is shown in Figures 9B,C, respectively. As shown in Figures 9A–C, the *D* + *Q* TBI group had a significantly greater NeuN+ cell number and reduced FJC intensity in all brain regions examined, as compared to the TBI + VH group. This finding suggests that senolytic therapy reduced neurodegeneration and neuron loss in the cortex, CA1, DG and LP of Thalamus long-term after TBI.

## Discussion

Understanding of the pathological processes and events that underlie chronic neuroinflammation after TBI has been hampered by a paucity of long-term studies. Only 10% of animal studies have examined timepoints greater than 2 months after TBI (Gold et al., 2013), and these focused primarily on functional endpoints with little attention to the underlying mechanisms. In the current study, we sought to address this deficit by performing long-term studies up to 4 months after TBI that examined both molecular and functional outcomes. Our study yielded several important findings. First, it confirmed a progressive, widespread neurodegeneration in the cortex, hippocampus CA1 region, dentate gyrus, and lateral posterior thalamus long-term after TBI, that was associated with a robust neuroinflammatory response, white matter damage, and a significant defect in cognitive function and depressive-like behavior. These findings are consistent with human studies that revealed neuroinflammatory events and white matter damage can persist in the brain many years after a single TBI (Ramlackhansingh et al., 2011; Johnson et al., 2013), and that nearly half of all hospitalized TBI survivors exhibit injury-related disabilities after one-year, including cognitive impairment, anxiety, and depression (Johnson et al., 2013).

Secondly, our study demonstrated a widespread induction of senescent cells in the ipsilateral cortex, hippocampus, CC and lateral posterior of the thalamus at 5 weeks and 4 months after TBI. Double immunohistochemistry demonstrated that the senescent cells were comprised of both astrocytes and microglia. This finding agrees with previous studies that identified senescent astrocytes and microglia in the injured cortex within days after TBI (Kozlova and Lukanidin, 2002; Ritzel et al., 2019; Tominaga et al., 2019), as well as in the aging and AD brain (Bhat et al., 2012; Bussian et al., 2018; Ogrodnik et al., 2021). Our finding of robust senescent cells in multiple regions in addition to the cortex weeks to several months after TBI, indicates a widespread induction of senescent cells occurs after TBI that persists long-term.

Intriguingly, recent work revealed that senescent astrocytes have a defect in their neuroprotective functions due to down-regulation of glutamate transporters (Limbad et al., 2020). Thus, a decrease in the neuroprotective function of senescent astrocytes could contribute to the robust neurodegeneration we observed in many brain areas long-term after TBI. Indeed, our study found that ablation of senescent astrocytes and microglia in the brain by senolytic therapy was associated with reduced neurodegeneration and enhanced neuron numbers in the ipsilateral cortex, hippocampal CA1 region, DG and LP of thalamus in TBI animals. Senescent cells also release SASP factors that can promote chronic neuroinflammation, and damage neighboring cells. Examination of the proinflammatory SASPs, IL-1β and IL-6 in our study revealed that both were significantly elevated in the ipsilateral cortex at 4 months after TBI, and this effect was markedly decreased by *D* + *Q* treatment. Therefore, in addition to reduction of the senescent cell burden, *D* + *Q* treatment also leads to a substantial reduction of the inflammatory (IL-1β and IL-6) burden in the brain long-term after TBI. While inflammation may be acutely beneficial, most studies suggest chronic, persistent inflammation is detrimental. In line with this suggestion, previous work has shown that sustained elevation of CSF IL-6 is associated with increased inflammatory load and increased risk of unfavorable outcomes in the first year following TBI in humans (Kumar et al., 2015). Furthermore, immunoneutralization of IL-1β has been shown to significantly reduce neuroinflammation and improve cognitive function after TBI (Lu et al., 2005; Clausen et al., 2011; Flygt et al., 2018).

Thirdly, in support of a potential role of senescent cells in long-term functional decline after TBI, our study demonstrated that delayed, intermittent *D* + *Q* senolytic treatment begun 1 month after TBI effectively ablated senescent cells and significantly rescued both the cognitive defect and depressive-like behavior in TBI mice at 4 months after TBI. Most animal studies on TBI initiate treatment either before TBI (pretreatment) or within 24–72 h (acute treatment) after TBI. Therefore, our study extends the therapeutic window significantly out to 1-month post TBI. Extending the therapeutic window in TBI is advantageous as it could provide a potential therapy to many existing patients who are long past the time of their TBI and currently have no therapeutic options.

The reason we chose *D* + *Q* for our study is that it is a first generation senolytic and is the most studied of all the senolytic drugs (Kirkland and Tchkonia, 2020). Furthermore, *D* + *Q* is an FDA approved drug/dietary supplement, which can quickly be repurposed for its senolytic ability for potentially faster translation to the clinic. The oral route and intermittent dosing schedule used in our study is consistent with the recommendations of an National Institute of Aging (NIA) Workshop on “Repurposing Drugs and Dietary Supplements for Senolytic or Senomorphic Consideration for Clinical Trials” (Romashkan et al., 2021). Translationally, an oral route for senolytic drug administration has several advantages, including its non-invasiveness, better patient compliance, and convenience of drug administration. Intermittent dosing has the important advantage that it minimizes the chance of any adverse effects of the drugs/supplements. Finally, it is important to note that intermittent oral administration of *D* + *Q* was recently shown to effectively decrease senescent cells in humans and to be well tolerated (Hickson et al., 2019; Nambiar et al., 2023).

While our study did not explore the mechanisms underlying senescent cell ablation by *D* + *Q*, previous work has shown that *D* + *Q* induces apoptosis in senescent, but not non-senescent cells in adipose, aorta, lungs, and brain (Zhu et al., 2015; Kirkland and Tchkonia, 2017; Bussian et al., 2018). Additional studies showed that D targets the “senescent cell associated anti-apoptotic” (SCAP) pathway, dependence receptors such as ephrins in part by inhibiting SRC kinase, while Q targets the BCL-2/BCL-XL, and p53/p21/serpine, and PI3k/Akt SCAPs (Kirkland and Tchkonia, 2017). The targeting of multiple SCAP pathways by the combination of *D* + *Q* together may explain its robust senolytic ability in this study, and in many other studies (Ogrodnik et al., 2019; Zhang et al., 2019; Ogrodnik et al., 2021; Budamagunta et al., 2023; Wang et al., 2023). It should be mentioned that our experimental approach has some limitations, including that oral *D* + *Q* targets the whole body. Thus, it is possible that clearance of senescent cells in the peripherally contributed to the beneficial effects observed in our study. However, it should be pointed out that both D and Q have been shown to cross the blood brain barrier (Porkka et al., 2008; Ishisaka et al., 2011; Zhang et al., 2019), and similar to our study, intermittent *D* + *Q* administration has been shown to ablate senescent cells and rescues cognitive function in animal models of both Alzheimer’s disease and aging (Zhang et al., 2019; Ogrodnik et al., 2021; Budamagunta et al., 2023). Nevertheless, additional studies are needed to address the question of central versus peripheral effects of *D* + *Q*. Still, the fact that *D* + *Q* can rescue cognitive function and depressive-like behavior when administered 1 month after TBI is significant. Another limitation is that we only examined male animals in our study and thus it is unclear if our findings extend to female animals. Few studies have examined senescent cells in the brain after TBI in female animals. The only study we are aware of in the literature used repetitive mild TBI (rTBI) and only examined senescent cell markers in the brain acutely (7 days after TBI) (Schwab et al., 2022). The study showed senescent cell markers are present in the female mouse brain at 7 days after rTBI, but surprisingly senolytic treatment with the drug ABT263 did not significantly reduce expression of the senescence cell markers. This lack of effect may be due to the fact that ABT263 only inhibits the BCL-2 family of senescence cell anti-apoptotic pathway (SCAP) network, and is ineffective against senescent cells that express multiple redundant SCAP networks (Wilson et al., 2010). Senescent cell ablation has been reported to be more effective if senolytic drugs like *D* + *Q* are used that target multiple SCAP networks (Kirkland and Tchkonia, 2020). Thus, in future studies, we plan to examine senescence cells in female animals both acutely and long-term after TBI and explore whether *D* + *Q* effectively ablates the senescent cells and enhances functional outcome, as observed in male animals in this study.”

While the current study results are encouraging, additional preclinical studies are needed to facilitate potential future translation to the clinic. For instance, it will be important in future studies to determine whether the therapeutic window can be extended even further past 1 month for *D* + *Q* benefit. It will also be important to determine whether the duration of *D* + *Q* administration can be reduced and still yield beneficial effect, and whether booster senolytic administration is needed at certain intervals to maintain senescent cell ablation. Our lab is actively pursuing the answers to these important questions.

With respect to clinical relevance of studies, our results suggest there may be broader implications for clinical applications and potential therapeutic options for TBI patients. For instance, activity of pro-inflammatory SASP factors, cytokines, and other markers could be monitored with ventriculostomy or microdialysis. Analytes could be followed over time for prognostication purposes or used to guide anti-inflammatory, immunomodulating, or senolytic treatment such as *D* + *Q*. The evolution of changes in the intracranial chemical environment after TBI could be correlated with radiographic, clinical, cognitive, and behavioral outcomes in human subjects. Answering these research questions related to broader implications would likewise require careful study of the long-term temporal pattern of senescent cell accumulation in the human brain after TBI, including determination of how long after TBI senolytic therapy can be started and still yield functional benefit. Our study findings suggest there may be practical benefit for TBI patients who present with some latency following their original injury, but this must be further investigated in human subjects.

In conclusion, the current study demonstrates that senescent cells are present weeks to months after TBI in multiple brain regions, and that ablation of the senescent cells and reduction of the SASP pro-inflammatory factors, IL-1β and IL-6, via administration of oral intermittent *D* + *Q* treatment beginning 1 month after TBI significantly reduces neurodegeneration and neuron loss in many brain areas, and improves both cognitive function and depressive-like behavior 4 months after TBI. These findings indicate that senescent cells may be an excellent therapeutic target in TBI, and that it may be possible to significantly extend the therapeutic window in TBI through senolytic therapy.

## Data availability statement

The raw data supporting the conclusions of this article will be made available by the authors, without undue reservation.

## Ethics statement

The animal study was reviewed and approved by Augusta University Institutional Animal Care and Use Committee.

## Author contributions

DB and KD contributed to conception and design of the study. DB was responsible for funding acquisition. YL, JW, and CC contributed to performance of the experiments and analysis of the data. DB wrote the first draft of the manuscript. YL and JW helped to wrote sections of the manuscript. All authors contributed to manuscript revision, read, and approved the submitted version.

## Funding

This work was supported by a research grant from the National Institute of Neurological Disorders and Stroke (1R01NS122724-01A1), National Institutes of Health.

## Conflict of interest

The authors declare that the research was conducted in the absence of any commercial or financial relationships that could be construed as a potential conflict of interest.

## Publisher’s note

All claims expressed in this article are solely those of the authors and do not necessarily represent those of their affiliated organizations, or those of the publisher, the editors and the reviewers. Any product that may be evaluated in this article, or claim that may be made by its manufacturer, is not guaranteed or endorsed by the publisher.

## Abbreviations

CCI: controlled cortical impact
TBI: traumatic brain injury

## References

[ref1] Baker D. J. Childs B. G. Durik M. Wijers M. E. Sieben C. J. Zhong J.. (2016). Naturally occurring p16(Ink4a)-positive cells shorten healthy lifespan. Nature 530, 184–189. doi: 10.1038/nature16932, PMID: 26840489 PMC4845101

[ref2] Baker D. J. Petersen R. C. (2018). Cellular senescence in brain aging and neurodegenerative diseases: evidence and perspectives. J. Clin. Invest. 128, 1208–1216. doi: 10.1172/JCI95145, PMID: 29457783 PMC5873891

[ref3] Baker D. J. Wijshake T. Tchkonia T. Lebrasseur N. K. Childs B. G. Van De Sluis B.. (2011). Clearance of p16Ink4a-positive senescent cells delays ageing-associated disorders. Nature 479, 232–236. doi: 10.1038/nature10600, PMID: 22048312 PMC3468323

[ref4] Bhat R. Crowe E. P. Bitto A. Moh M. Katsetos C. D. Garcia F. U.. (2012). Astrocyte senescence as a component of Alzheimer's disease. PLoS One 7:e45069. doi: 10.1371/journal.pone.0045069, PMID: 22984612 PMC3440417

[ref5] Budamagunta V. Kumar A. Rani A. Bean L. Manohar-Sindhu S. Yang Y.. (2023). Effect of peripheral cellular senescence on brain aging and cognitive decline. Aging Cell 22:e13817. doi: 10.1111/acel.13817 36959691 PMC10186609

[ref6] Bussian T. J. Aziz A. Meyer C. F. Swenson B. L. Van Deursen J. M. Baker D. J. (2018). Clearance of senescent glial cells prevents tau-dependent pathology and cognitive decline. Nature 562, 578–582. doi: 10.1038/s41586-018-0543-y, PMID: 30232451 PMC6206507

[ref7] Childs B. G. Gluscevic M. Baker D. J. Laberge R. M. Marquess D. Dananberg J.. (2017). Senescent cells: an emerging target for diseases of ageing. Nat. Rev. Drug Discov. 16, 718–735. doi: 10.1038/nrd.2017.116, PMID: 28729727 PMC5942225

[ref8] Chinta S. J. Woods G. Rane A. Demaria M. Campisi J. Andersen J. K. (2015). Cellular senescence and the aging brain. Exp. Gerontol. 68, 3–7. doi: 10.1016/j.exger.2014.09.018 25281806 PMC4382436

[ref9] Clausen F. Hanell A. Israelsson C. Hedin J. Ebendal T. Mir A. K.. (2011). Neutralization of interleukin-1beta reduces cerebral edema and tissue loss and improves late cognitive outcome following traumatic brain injury in mice. Eur. J. Neurosci. 34, 110–123. doi: 10.1111/j.1460-9568.2011.07723.x, PMID: 21623956

[ref10] Cohen J. Torres C. (2019). Astrocyte senescence: evidence and significance. Aging Cell 18:e12937. doi: 10.1111/acel.12937, PMID: 30815970 PMC6516680

[ref11] Debacq-Chainiaux F. Erusalimsky J. D. Campisi J. Toussaint O. (2009). Protocols to detect senescence-associated beta-galactosidase (SA-betagal) activity, a biomarker of senescent cells in culture and in vivo. Nat. Protoc. 4, 1798–1806. doi: 10.1038/nprot.2009.191, PMID: 20010931

[ref12] Faden A. I. Wu J. Stoica B. A. Loane D. J. (2016). Progressive inflammation-mediated neurodegeneration after traumatic brain or spinal cord injury. Br. J. Pharmacol. 173, 681–691. doi: 10.1111/bph.13179, PMID: 25939377 PMC4742301

[ref13] Flygt J. Ruscher K. Norberg A. Mir A. Gram H. Clausen F.. (2018). Neutralization of interleukin-1beta following diffuse traumatic brain injury in the mouse attenuates the loss of mature oligodendrocytes. J. Neurotrauma 35, 2837–2849. doi: 10.1089/neu.2018.5660, PMID: 29690837 PMC6247990

[ref14] Gold E. M. Su D. Lopez-Velazquez L. Haus D. L. Perez H. Lacuesta G. A.. (2013). Functional assessment of long-term deficits in rodent models of traumatic brain injury. Regen. Med. 8, 483–516. doi: 10.2217/rme.13.41 23826701

[ref15] Hickson L. J. Langhi Prata L. G. P. Bobart S. A. Evans T. K. Giorgadze N. Hashmi S. K.. (2019). Senolytics decrease senescent cells in humans: preliminary report from a clinical trial of Dasatinib plus Quercetin in individuals with diabetic kidney disease. EBioMedicine 47, 446–456. doi: 10.1016/j.ebiom.2019.08.069, PMID: 31542391 PMC6796530

[ref16] Hoover R. C. Motta M. Davis J. Saatman K. E. Fujimoto S. T. Thompson H. J.. (2004). Differential effects of the anticonvulsant topiramate on neurobehavioral and histological outcomes following traumatic brain injury in rats. J. Neurotrauma 21, 501–512. doi: 10.1089/089771504774129847, PMID: 15165359

[ref17] Ishisaka A. Ichikawa S. Sakakibara H. Piskula M. K. Nakamura T. Kato Y.. (2011). Accumulation of orally administered quercetin in brain tissue and its antioxidative effects in rats. Free Radic. Biol. Med. 51, 1329–1336. doi: 10.1016/j.freeradbiomed.2011.06.017, PMID: 21741473

[ref18] Johnson V. E. Stewart J. E. Begbie F. D. Trojanowski J. Q. Smith D. H. Stewart W. (2013). Inflammation and white matter degeneration persist for years after a single traumatic brain injury. Brain 136, 28–42. doi: 10.1093/brain/aws322, PMID: 23365092 PMC3562078

[ref19] Kirkland J. L. Tchkonia T. (2017). Cellular senescence: a translational perspective. EBioMedicine 21, 21–28. doi: 10.1016/j.ebiom.2017.04.013, PMID: 28416161 PMC5514381

[ref20] Kirkland J. L. Tchkonia T. (2020). Senolytic drugs: from discovery to translation. J. Intern. Med. 288, 518–536. doi: 10.1111/joim.13141, PMID: 32686219 PMC7405395

[ref21] Kozlova E. N. Lukanidin E. (2002). Mts1 protein expression in the central nervous system after injury. Glia 37, 337–348. doi: 10.1002/glia.10045, PMID: 11870873

[ref22] Kritsilis M. Rizou S. V. Koutsoudaki P. Evangelou K. Gorgoulis V. Papadopoulos D. (2018). Ageing, cellular senescence and neurodegenerative disease. Int. J. Mol. Sci. 19:2937. doi: 10.3390/ijms19102937, PMID: 30261683 PMC6213570

[ref23] Kumar R. G. Diamond M. L. Boles J. A. Berger R. P. Tisherman S. A. Kochanek P. M.. (2015). Acute CSF interleukin-6 trajectories after TBI: associations with neuroinflammation, polytrauma, and outcome. Brain Behav. Immun. 45, 253–262. doi: 10.1016/j.bbi.2014.12.021, PMID: 25555531

[ref24] Laird M. D. Sukumari-Ramesh S. Swift A. E. Meiler S. E. Vender J. R. Dhandapani K. M. (2010). Curcumin attenuates cerebral edema following traumatic brain injury in mice: a possible role for aquaporin-4? J. Neurochem. 113, 637–648. doi: 10.1111/j.1471-4159.2010.06630.x, PMID: 20132469 PMC2911034

[ref25] Li B. Mahmood A. Lu D. Wu H. Xiong Y. Qu C.. (2009). Simvastatin attenuates microglial cells and astrocyte activation and decreases interleukin-1beta level after traumatic brain injury. Neurosurgery 65:179–185. doi: 10.1227/01.NEU.0000346272.76537.DC 19574840 PMC2749520

[ref26] Limbad C. Oron T. R. Alimirah F. Davalos A. R. Tracy T. E. Gan L.. (2020). Astrocyte senescence promotes glutamate toxicity in cortical neurons. PLoS One 15:e0227887. doi: 10.1371/journal.pone.0227887, PMID: 31945125 PMC6964973

[ref27] Loane D. J. Kumar A. Stoica B. A. Cabatbat R. Faden A. I. (2014). Progressive neurodegeneration after experimental brain trauma: association with chronic microglial activation. J. Neuropathol. Exp. Neurol. 73, 14–29. doi: 10.1097/NEN.0000000000000021, PMID: 24335533 PMC4267248

[ref28] Lu Y. Dong Y. Tucker D. Wang R. Ahmed M. E. Brann D.. (2017). Treadmill exercise exerts neuroprotection and regulates microglial polarization and oxidative stress in a streptozotocin-induced rat model of sporadic Alzheimer's disease. J. Alzheimers Dis. 56, 1469–1484. doi: 10.3233/JAD-160869, PMID: 28157094 PMC5450951

[ref29] Lu Y. Sareddy G. R. Wang J. Wang R. Li Y. Dong Y.. (2019). Neuron-derived estrogen regulates synaptic plasticity and memory. J. Neurosci. 39, 2792–2809. doi: 10.1523/JNEUROSCI.1970-18.2019, PMID: 30728170 PMC6462452

[ref30] Lu Y. Sareddy G. R. Wang J. Zhang Q. Tang F. L. Pratap U. P.. (2020). Neuron-derived estrogen is critical for astrocyte activation and neuroprotection of the ischemic brain. J. Neurosci. 40, 7355–7374. doi: 10.1523/JNEUROSCI.0115-20.2020, PMID: 32817249 PMC7534920

[ref31] Lu K. T. Wang Y. W. Yang J. T. Yang Y. L. Chen H. I. (2005). Effect of interleukin-1 on traumatic brain injury-induced damage to hippocampal neurons. J. Neurotrauma 22, 885–895. doi: 10.1089/neu.2005.22.885, PMID: 16083355

[ref32] Ma M. W. Wang J. Dhandapani K. M. Brann D. W. (2017). NADPH oxidase 2 regulates NLRP3 inflammasome activation in the brain after traumatic brain injury. Oxidative Med. Cell. Longev. 2017, 1–18. doi: 10.1155/2017/6057609 PMC5529650 28785377

[ref33] Maas A. I. R. Menon D. K. Adelson P. D. Andelic N. Bell M. J. Belli A.. (2017). Traumatic brain injury: integrated approaches to improve prevention, clinical care, and research. Lancet Neurol. 16, 987–1048. doi: 10.1016/S1474-4422(17)30371-X, PMID: 29122524

[ref34] Nambiar A. Kellogg D. 3rd Justice J. Goros M. Gelfond J. Pascual R.. (2023). Senolytics dasatinib and quercetin in idiopathic pulmonary fibrosis: results of a phase I, single-blind, single-center, randomized, placebo-controlled pilot trial on feasibility and tolerability. EBioMedicine 90:104481. doi: 10.1016/j.ebiom.2023.104481, PMID: 36857968 PMC10006434

[ref35] Ng P. Y. Mcneely T. L. Baker D. J. (2023). Untangling senescent and damage-associated microglia in the aging and diseased brain. FEBS J. 290, 1326–1339. doi: 10.1111/febs.16315, PMID: 34873840 PMC9167891

[ref36] Ogrodnik M. Evans S. A. Fielder E. Victorelli S. Kruger P. Salmonowicz H.. (2021). Whole-body senescent cell clearance alleviates age-related brain inflammation and cognitive impairment in mice. Aging Cell 20:e13296. doi: 10.1111/acel.13296 33470505 PMC7884042

[ref37] Ogrodnik M. Zhu Y. Langhi L. G. P. Tchkonia T. Kruger P. Fielder E.. (2019). Obesity-induced cellular senescence drives anxiety and impairs neurogenesis. Cell Metab. 29:1233. doi: 10.1016/j.cmet.2019.01.013, PMID: 31067450 PMC6509279

[ref38] Porkka K. Koskenvesa P. Lundan T. Rimpilainen J. Mustjoki S. Smykla R.. (2008). Dasatinib crosses the blood-brain barrier and is an efficient therapy for central nervous system Philadelphia chromosome-positive leukemia. Blood 112, 1005–1012. doi: 10.1182/blood-2008-02-140665 18477770

[ref39] Ramlackhansingh A. F. Brooks D. J. Greenwood R. J. Bose S. K. Turkheimer F. E. Kinnunen K. M.. (2011). Inflammation after trauma: microglial activation and traumatic brain injury. Ann. Neurol. 70, 374–383. doi: 10.1002/ana.22455 21710619

[ref40] Ritzel R. M. Doran S. J. Glaser E. P. Meadows V. E. Faden A. I. Stoica B. A.. (2019). Old age increases microglial senescence, exacerbates secondary neuroinflammation, and worsens neurological outcomes after acute traumatic brain injury in mice. Neurobiol. Aging 77, 194–206. doi: 10.1016/j.neurobiolaging.2019.02.010, PMID: 30904769 PMC6486858

[ref41] Romashkan S. Chang H. Hadley E. C. (2021). National Institute on Aging workshop: repurposing drugs or dietary supplements for their Senolytic or Senomorphic effects: considerations for clinical trials. J. Gerontol. A Biol. Sci. Med. Sci. 76, 1144–1152. doi: 10.1093/gerona/glab028, PMID: 33528569 PMC8521777

[ref42] Saccon T. D. Nagpal R. Yadav H. Cavalcante M. B. Nunes A. D. C. Schneider A.. (2021). Senolytic combination of Dasatinib and Quercetin alleviates intestinal senescence and inflammation and modulates the gut microbiome in aged mice. J. Gerontol. A Biol. Sci. Med. Sci. 76, 1895–1905. doi: 10.1093/gerona/glab002, PMID: 33406219 PMC8514064

[ref43] Salminen A. Ojala J. Kaarniranta K. Haapasalo A. Hiltunen M. Soininen H. (2011). Astrocytes in the aging brain express characteristics of senescence-associated secretory phenotype. Eur. J. Neurosci. 34, 3–11. doi: 10.1111/j.1460-9568.2011.07738.x, PMID: 21649759

[ref44] Schwab N. Grenier K. Hazrati L. N. (2019). DNA repair deficiency and senescence in concussed professional athletes involved in contact sports. Acta Neuropathol. Commun. 7:182. doi: 10.1186/s40478-019-0822-3, PMID: 31727161 PMC6857343

[ref45] Schwab N. Ju Y. Hazrati L. N. (2021). Early onset senescence and cognitive impairment in a murine model of repeated mTBI. Acta Neuropathol. Commun. 9:82. doi: 10.1186/s40478-021-01190-x, PMID: 33964983 PMC8106230

[ref46] Schwab N. Taskina D. Leung E. Innes B. T. Bader G. D. Hazrati L. N. (2022). Neurons and glial cells acquire a senescent signature after repeated mild traumatic brain injury in a sex-dependent manner. Front. Neurosci. 16:1027116. doi: 10.3389/fnins.2022.1027116, PMID: 36408415 PMC9669743

[ref47] Simon D. W. Mcgeachy M. J. Bayir H. Clark R. S. Loane D. J. Kochanek P. M. (2017). The far-reaching scope of neuroinflammation after traumatic brain injury. Nat. Rev. Neurol. 13, 171–191. doi: 10.1038/nrneurol.2017.13, PMID: 28186177 PMC5675525

[ref48] Tchkonia T. Zhu Y. Van Deursen J. Campisi J. Kirkland J. L. (2013). Cellular senescence and the senescent secretory phenotype: therapeutic opportunities. J. Clin. Invest. 123, 966–972. doi: 10.1172/JCI64098, PMID: 23454759 PMC3582125

[ref49] Thompson H. J. Marklund N. Lebold D. G. Morales D. M. Keck C. A. Vinson M.. (2006). Tissue sparing and functional recovery following experimental traumatic brain injury is provided by treatment with an anti-myelin-associated glycoprotein antibody. Eur. J. Neurosci. 24, 3063–3072. doi: 10.1111/j.1460-9568.2006.05197.x, PMID: 17156367 PMC2377452

[ref50] Tominaga T. Shimada R. Okada Y. Kawamata T. Kibayashi K. (2019). Senescence-associated-beta-galactosidase staining following traumatic brain injury in the mouse cerebrum. PLoS One 14:e0213673. doi: 10.1371/journal.pone.0213673, PMID: 30856215 PMC6411151

[ref51] Tuzer F. Torres C. (2022). Involvement of astrocyte senescence in Alzheimer's disease. Curr. Opin. Neurobiol. 76:102594. doi: 10.1016/j.conb.2022.102594 35779313

[ref52] Wang C. Kang Y. Liu P. Liu W. Chen W. Hayashi T.. (2023). Combined use of dasatinib and quercetin alleviates overtraining-induced deficits in learning and memory through eliminating senescent cells and reducing apoptotic cells in rat hippocampus. Behav. Brain Res. 440:114260. doi: 10.1016/j.bbr.2022.114260, PMID: 36535433

[ref53] Wang J. Ma M. W. Dhandapani K. M. Brann D. W. (2017). Regulatory role of NADPH oxidase 2 in the polarization dynamics and neurotoxicity of microglia/macrophages after traumatic brain injury. Free Radic. Biol. Med. 113, 119–131. doi: 10.1016/j.freeradbiomed.2017.09.017, PMID: 28942245

[ref54] Wang J. Sareddy G. R. Lu Y. Pratap U. P. Tang F. Greene K. M.. (2020). Astrocyte-derived estrogen regulates reactive astrogliosis and is neuroprotective following ischemic brain injury. J. Neurosci. 40, 9751–9771. doi: 10.1523/JNEUROSCI.0888-20.2020, PMID: 33158962 PMC7726540

[ref55] Wilson W. H. O'connor O. A. Czuczman M. S. Lacasce A. S. Gerecitano J. F. Leonard J. P.. (2010). Navitoclax, a targeted high-affinity inhibitor of BCL-2, in lymphoid malignancies: a phase 1 dose-escalation study of safety, pharmacokinetics, pharmacodynamics, and antitumour activity. Lancet Oncol. 11, 1149–1159. doi: 10.1016/S1470-2045(10)70261-8, PMID: 21094089 PMC3025495

[ref56] Xiong Y. Mahmood A. Chopp M. (2018). Current understanding of neuroinflammation after traumatic brain injury and cell-based therapeutic opportunities. Chin. J. Traumatol. 21, 137–151. doi: 10.1016/j.cjtee.2018.02.003, PMID: 29764704 PMC6034172

[ref57] Zhang P. Kishimoto Y. Grammatikakis I. Gottimukkala K. Cutler R. G. Zhang S.. (2019). Senolytic therapy alleviates Abeta-associated oligodendrocyte progenitor cell senescence and cognitive deficits in an Alzheimer's disease model. Nat. Neurosci. 22, 719–728. doi: 10.1038/s41593-019-0372-9, PMID: 30936558 PMC6605052

[ref58] Zhang Q. G. Laird M. D. Han D. Nguyen K. Scott E. Dong Y.. (2012). Critical role of NADPH oxidase in neuronal oxidative damage and microglia activation following traumatic brain injury. PLoS One 7:e34504. doi: 10.1371/journal.pone.0034504, PMID: 22485176 PMC3317633

[ref59] Zhu Y. Tchkonia T. Pirtskhalava T. Gower A. C. Ding H. Giorgadze N.. (2015). The Achilles' heel of senescent cells: from transcriptome to senolytic drugs. Aging Cell 14, 644–658. doi: 10.1111/acel.12344, PMID: 25754370 PMC4531078

