# Supplementary material for: Senolytic therapy is neuroprotective and improves functional outcome long-term after traumatic brain injury in mice
Source: Front Neurosci. 2023 Jul 27;17:1227705. doi: 10.3389/fnins.2023.1227705 (PMC10416099; doi:10.3389/fnins.2023.1227705)
Supplement: Supplementary file 1 [file Table_1.DOCX]

| **Figure** | **ANOVA** | **Result** |
| --- | --- | --- |
| **Figure 1B** | One way ANOVA | F (3, 27) = 21.06 P<0.0001 |
| **Figure 1C** | One way ANOVA | F (3, 27) = 59.26 P<0.0001 |
| **Figure 1F** | Two Way ANOVA | Interaction F (3, 40) = 7.935 P=0.0003  Row Factor F (3, 40) = 7.877 P=0.0003  Column factor F (1, 40) = 113.3 P<0.0001 |
| **Figure 1G** | Two way ANOVA | Interaction F (3, 40) = 40.19 P<0.0001  Row factor F (3, 40) = 40.21 P<0.0001  Column factor F (1, 40) = 540.6 P<0.0001 |
| **Figure 2A (b)** | Two way ANOVA | Interaction F (2, 33) = 1.189 P=0.3171  Row factor F (2, 33) = 10.87 P=0.0002  Column factor F (1, 33) = 13.68 P=0.0008 |
| **Figure 2C (b)** | Two way ANOVA | Interaction F (1, 24) = 0.01937 P=0.8905  Row factor F (1, 24) = 0.03797 P=0.8472  Column factor F (1, 24) = 1.827 P=0.1891 |
| **Figure 2C (c)** | Two way ANOVA | Interaction F (1, 24) = 9.761 P=0.0046  Row factor F (1, 24) = 2.785 P=0.1082  Column factor F (1, 24) = 4.490 P=0.0446 |
| **Figure 3B** | One way ANOVA | Cortex F (2, 15) = 273.5 P<0.0001  CA1 F (2, 15) = 92.95 P<0.0001  DG F (2, 15) = 125.0 P<0.0001  CC F (2, 15) = 53.45 P<0.0001  LP F (2, 15) = 302.3 P<0.0001 |
| **Figure 4E** | Two way ANOVA | Interaction F (2, 30) = 30.42 P<0.0001  Row factor F (1, 30) = 6.367 P=0.0172  Column factor F (2, 30) = 156.9 P<0.0001 |
| **Figure 5E** | One way ANOVA | Cortex F (2, 15) = 151.4 P<0.0001  CA1 F (2, 15) = 48.21 P<0.0001  DG F (2, 15) = 55.90 P<0.0001  CC F (2, 15) = 203.6 P<0.0001  LP F (2, 15) = 207.9 P<0.0001 |
| **Figure 5F** | One way ANOVA | Cortex F (2, 15) = 106.7 P<0.0001  CA1 F (2, 15) = 7.000 P=0.0071  DG F (2, 15) = 95.89 P<0.0001  CC F (2, 15) = 127.9 P<0.0001  LP F (2, 15) = 150.1 P<0.0001 |
| **Figure 6A (b)** | Two way ANOVA-Cortex | Interaction F (1, 20) = 75.56 P<0.0001  Row factor F (1, 20) = 313.8 P<0.0001  Column factor F (1, 20) = 75.56 P<0.0001 |
|  | Two way ANOVA-CC | Interaction F (1, 20) = 121.3 P<0.0001  Row factor F (1, 20) = 275.7 P<0.0001  Column factor F (1, 20) = 121.3 P<0.0001 |
|  | Two way ANOVA-CA1 | Interaction F (1, 20) = 86.43 P<0.0001  Row Factor (1, 20) = 1086 P<0.0001  Column factor F (1, 20) = 86.43 P<0.0001 |
|  | Two way ANOVA-DG | Interaction F (1, 20) = 160.7 P<0.0001  Row factor F (1, 20) = 1143 P<0.0001  Column factor F (1, 20) = 160.7 P<0.0001 |
|  | Two way ANOVA-CALP | Interaction F (1, 20) = 121.6 P<0.0001  Row factor F (1, 20) = 851.1 P<0.0001  Column factor F (1, 20) = 121.6 P<0.0001 |
| **Figure 6B (b)** | Two way ANOVA-Cortex | Interaction F (1, 20) = 135.0 P<0.0001  Row factor F (1, 20) = 960.0 P<0.0001  Column factor F (1, 20) = 135.0 P<0.0001 |
|  | Two way ANOVA-CC | Interaction F (1, 20) = 100.5 P<0.0001  Row factor F (1, 20) = 720.1 P<0.0001  Column factor F (1, 20) = 100.5 P<0.0001 |
|  | Two way ANOVA-CA1 | Interaction F (1, 20) = 81.77 P<0.0001  Row factor F (1, 20) = 526.9 P<0.0001  Column factor F (1, 20) = 81.77 P<0.0001 |
|  | Two way ANOVA-DG | Interaction F (1, 20) = 167.0 P<0.0001  Row factor F (1, 20) = 1101 P<0.0001  Column factor F (1, 20) = 167.0 P<0.0001 |
|  | Two way ANOVA-LP | \| Interaction F (1, 20) = 48.08 \| P<0.0001 \| \| --- \| --- \| \| Row factor F (1, 20) = 345.3 \| P<0.0001 \| \| Column factor F (1, 20) = 48.08 \| P<0.0001 \| |
| **Figure 6C (c)** | Two way ANOVA | Interaction F (1, 20) = 10.42 P=0.0042  Row factor F (1, 20) = 20.44 P=0.0002  Column factor F (1, 20) = 83.93 P<0.0001 |
| **Figure 6C (d)** | Two way ANOVA | Interaction F (1, 20) = 41.82 P<0.0001  Row factor F (1, 20) = 70.94 P<0.0001  Column factor F (1, 20) = 155.0 P<0.0001 |
| **Figure 6C (e)** | Two way ANOVA | Interaction F (1, 20) = 14.05 P=0.0013  Row factor F (1, 20) = 16.74 P=0.0006  Column factor F (1, 20) = 34.13 P<0.0001 |
| **Figure 6D** | Two way ANOVA | Interaction F (1, 20) = 4.646 P=0.0435  Row factor F (1, 20) = 12.02 P=0.0024  Column factor F (1, 20) = 31.53 P<0.0001 |
| **Figure 7A (b)** | Two way ANOVA-Cortex | Interaction F (1, 20) = 5.462 P=0.0476  Row factor F (1, 20) = 98.46 P<0.0001  Column factor F (1, 20) = 31.15 P<0.0001 |
|  | Two way ANOVA-CC | Interaction F (1, 20) = 77.16 P<0.0001  Row factor F (1, 20) = 587.8 P<0.0001  Column factor F (1, 20) = 83.46 P<0.0001 |
|  | Two way ANOVA-CA1 | Interaction F (1, 20) = 34.29 P<0.0001  Row factor F (1, 20) = 137.1 P<0.0001  Column factor F (1, 20) = 34.29 P<0.0001 |
|  | Two way ANOVA-DG | Interaction F (1, 20) = 11.75 P=0.0027  Row factor F (1, 20) = 123.0 P<0.0001  Column factor F (1, 20) = 29.63 P<0.0001 |
|  | Two way ANOVA-LP | Interaction F (1, 20) = 258.9 P<0.0001  Row factor F (1, 20) = 890.0 P<0.0001  Column factor F (1, 20) = 280.9 P<0.0001 |
| **Figure 7B (b)** | Two way ANOVA-Cortex | Interaction F (1, 20) = 21.50 P=0.0002  Row factor F (1, 20) = 193.5 P<0.0001  Column factor F (1, 20) = 34.19 P<0.0001 |
|  | Two way ANOVA-CC | Interaction F (1, 20) = 120.1 P<0.0001  Row factor F (1, 20) = 426.0 P<0.0001  Column factor F (1, 20) = 168.9 P<0.0001 |
|  | Two way ANOVA-CA1 | Interaction F (1, 20) = 34.35 P<0.0001  Row factor F (1, 20) = 143.4 P<0.0001  Column factor F (1, 20) = 54.61 P<0.0001 |
|  | Two way ANOVA-DG | Interaction F (1, 20) = 14.93 P=0.0010  Row factor F (1, 20) = 38.21 P<0.0001  Column factor F (1, 20) = 25.22 P<0.0001 |
|  | Two way ANOVA-LP | Interaction F (1, 20) = 72.73 P<0.0001  Row factor F (1, 20) = 676.5 P<0.0001  Column factor F (1, 20) = 210.2 P<0.0001 |
| **Figure 8A (e)** | Two way ANOVA | Interaction F (1, 28) = 13.235 P=0.0008  Row Factor F (1, 28) = 17.825 P=0.0006  Column Factor F (1, 28) = 7.011 P=0.022 |
| **Figure 8A (f)** | Two way ANOVA | Interaction F (1, 28) = 7.075 P=0.020  Row Factor F (1, 28) = 6.314 P=0.038  Column Factor F (1, 28) = 6.907 P=0.026 |
| **Figure 8A (g)** | Two way ANOVA | Interaction F (1, 28) = 0.06524 P=0.8003  Row Factor F (1, 28) = 0.7991 P=0.3790  Column Factor F (1, 28) = 0.0002013 P=0.9888 |
| **Figure 8B (d)** | Two way ANOVA | Interaction F (1, 28) = 12.143 P=0.0015  Row Factor F (1, 28) = 7.117 P=0.0125  Column Factor F (1, 28) = 11.15 P=0.0024 |
| **Figure 8C** | Two way ANOVA | Interaction F (1, 28) = 10.10 P=0.0036  Row Factor F (1, 28) = 9.916 P=0.0039  Column Factor F (1, 28) = 25.27 P<0.0001 |
| **Figure 9B** | Two way ANOVA-Cortex | Interaction F (1, 20) = 7.937 P=0.0106  Row factor F (1, 20) = 29.79 P<0.0001  Column factor F (1, 20) = 6.669 P=0.0178 |
|  | Two way ANOVA-CA1 | Interaction F (1, 20) = 21.79 P=0.0001  Row factor F (1, 20) = 79.30 P<0.0001  Column factor F (1, 20) = 16.97 P=0.0005 |
|  | Two way ANOVA-DG | Interaction F (1, 20) = 13.00 P=0.0018  Row factor F (1, 20) = 160.2 P<0.0001  Column factor F (1, 20) = 21.62 P=0.0002 |
|  | Two way ANOVA-LP | Interaction F (1, 20) = 11.15 P=0.0033  Row factor F (1, 20) = 93.25 P<0.0001  Column factor F (1, 20) = 17.73 P=0.0004 |
| **Figure 9C** | Two way ANOVA-Cortex | Interaction F (1, 20) = 36.95 P<0.0001  Row factor F (1, 20) = 468.9 P<0.0001  Column factor F (1, 20) = 34.94 P<0.0001 |
|  | Two way ANOVA-CA1 | Interaction F (1, 20) = 28.95 P<0.0001  Row factor F (1, 20) = 476.8 P<0.0001  Column factor F (1, 20) = 27.90 P<0.0001 |
|  | Two way ANOVA-DG | Interaction F (1, 20) = 85.75 P<0.0001  Row factor F (1, 20) = 493.9 P<0.0001  Column factor F (1, 20) = 90.91 P<0.0001 |
|  | Two way ANOVA-LP | Interaction F (1, 20) = 53.53 P<0.0001  Row factor F (1, 20) = 504.0 P<0.0001  Column factor F (1, 20) = 50.44 P<0.0001 |
